# Gene Expression and Biological Pathways in Tissue of Men with Prostate Cancer in a Randomized Clinical Trial of Lycopene and Fish Oil Supplementation

**DOI:** 10.1371/journal.pone.0024004

**Published:** 2011-09-01

**Authors:** Mark Jesus M. Magbanua, Ritu Roy, Eduardo V. Sosa, Vivian Weinberg, Scott Federman, Michael D. Mattie, Millie Hughes-Fulford, Jeff Simko, Katsuto Shinohara, Christopher M. Haqq, Peter R. Carroll, June M. Chan

**Affiliations:** 1 Helen Diller Family Comprehensive Cancer Center, University of California San Francisco, San Francisco, California, United States of America; 2 Helen Diller Family Comprehensive Cancer Center Biostatistics and Computational Biology Core, University of California San Francisco, San Francisco, California, United States of America; 3 Department of Urology, University of California San Francisco, San Francisco, California, United States of America; 4 San Francisco Veterans Affairs Medical Center, San Francisco, California, United States of America; 5 Department of Pathology, University of California San Francisco, San Francisco, California, United States of America; 6 Department of Epidemiology and Biostatistics, University of California San Francisco, San Francisco, California, United States of America; Roswell Park Cancer Institute, United States of America

## Abstract

**Background:**

Studies suggest that micronutrients may modify the risk or delay progression of prostate cancer; however, the molecular mechanisms involved are poorly understood. We examined the effects of lycopene and fish oil on prostate gene expression in a double-blind placebo-controlled randomized clinical trial.

**Methods:**

Eighty-four men with low risk prostate cancer were stratified based on self-reported dietary consumption of fish and tomatoes and then randomly assigned to a 3-month intervention of lycopene (n = 29) or fish oil (n = 27) supplementation or placebo (n = 28). Gene expression in morphologically normal prostate tissue was studied at baseline and at 3 months via cDNA microarray analysis. Differential gene expression and pathway analyses were performed to identify genes and pathways modulated by these micronutrients.

**Results:**

Global gene expression analysis revealed no significant individual genes that were associated with high intake of fish or tomato at baseline or after 3 months of supplementation with lycopene or fish oil. However, exploratory pathway analyses of rank-ordered genes (based on p-values not corrected for multiple comparisons) revealed the modulation of androgen and estrogen metabolism in men who routinely consumed more fish (p = 0.029) and tomato (p = 0.008) compared to men who ate less. In addition, modulation of arachidonic acid metabolism (p = 0.01) was observed after 3 months of fish oil supplementation compared with the placebo group; and modulation of nuclear factor (erythroid derived-2) factor 2 or *Nrf2*-mediated oxidative stress response for either supplement versus placebo (fish oil: p = 0.01, lycopene: p = 0.001).

**Conclusions:**

We did not detect significant individual genes associated with dietary intake and supplementation of lycopene and fish oil. However, exploratory analyses revealed candidate *in vivo* pathways that may be modulated by these micronutrients.

**Trial Registration:**

ClinicalTrials.gov NCT00402285

## Introduction

Many men with indolent prostate cancer detected by prostate-specific antigen (PSA) screening will not exhibit disease progression during their lifetime. As such, their treatment and associated side effects may be unnecessary [1]. Active surveillance is a management strategy that offers close monitoring of localized cancers to avoid or delay the comorbidity of invasive treatments such as surgery or radiation [2]. Therapeutic intervention is offered at early signs of disease progression. Recently, the investigation of dietary and lifestyle interventions and other chemoprevention strategies in the setting of active surveillance has gained considerable acceptance [3]. For example, our group recently reported the results of a pilot project involving 30 men who underwent a three month intervention of a low-fat vegan diet and comprehensive changes in lifestyle [4]. Expression profiling and pathway analysis identified significant down regulation of genes involved in biological processes that have critical roles in tumorigenesis.

Laboratory and animal experiments provide evidence that fish oil [5] and lycopene [6] may play a protective role in prostate cancer development. In addition, epidemiological studies have shown that diets rich in fish and tomatoes, which are major sources of dietary omega-3 fatty acids and lycopene, respectively, are associated with a lower incidence of prostate cancer [7], [8], [9], [10], [11]. We (Chan JM) [12] previously reported that greater intakes of fish and tomato sauce after diagnosis of prostate cancer were associated with a reduction in risk of prostate cancer recurrence or progression in a cohort of 1202 prostate cancer survivors. While these studies link lycopene and fish oil with reduced prostate cancer progression risk, the molecular mechanisms of action of these dietary factors have yet to be elucidated.

To understand the effects of lycopene and fish oil on prostate gene expression, we conducted a randomized, double-blinded, placebo-controlled clinical trial among men with newly diagnosed, favorable-risk prostate cancer (MENS or Molecular Effects of Nutrition Supplements). Eligible men were randomized to take placebo, lycopene or fish oil supplements for three months. The trial's original hypotheses focused on quantitative RT-PCR analysis of *a priori* genes of interest (*IGF-1, IGF-1R, COX-2*). The results of this analysis were primarily null and have been reported separately [13]. We now report on the *a priori* secondary outcomes of global gene expression and pathway analyses of morphologically normal prostate tissue before and after the intervention.

## Methods

### Study Design

The study design and rationale for MENS has been reported previously [13]. The protocol for this trial and supporting CONSORT checklist are available as supporting information; see Checklist S1 and Protocol S1. Briefly, MENS was a three arm randomized, double blinded placebo-controlled 90 day clinical trial of fish oil and lycopene supplements with each compared to a placebo. The interventions consisted of two 15 mg lycopene softgel capsules daily (Lyc-O-Mato® donated by, Lycored, Israel) or three 1 gm fish oil capsules daily (that included 1098 mg eicosapentaenoic (EPA) and 549 mg docosahexaenoic (DHA) fatty acids; manufactured by Perfect Source, Fullerton, CA with active ingredient donated by Roche Vitamins, Parsippany, NJ). The placebo was provided by the respective manufacturers of the active pills for lycopene and fish oil. All men were also given a standard multivitamin (1 tablet/day, Dixon/Akyma) and instructed to refrain from any other types of vitamin or mineral supplements during the three months of intervention. The fish oil and lycopene doses were selected based on the minimal and maximal range at which previous clinical trials had observed physiologic effects and no toxicity for lycopene [14], [15], [16], [17], [18] [19] [7], [20] and fish oil supplementation [21], [22], [23], [24], [25], [26], [27]. The intervention duration of three months was chosen based on previous studies that reported on gene expression changes within three months [15], [21], [28]; and based on consultation with local prostate cancer advocates and support groups who advised that most men would prefer not to stop other supplements for more than three-months.

Baseline dietary data on fish and tomato consumption was used for stratification (i.e., more than 4 servings/day tomato products = high; more than 2 servings/week fish = high; cut points based on the published literature [12]) with patients randomized from within each stratum to control for possible confounding when comparing the intervention arms. The randomization sequence for the 4 nutrition strata was generated using nQuery Advisor with a block size of 9. For each stratum a sequence of randomized assignments was generated and given to the research pharmacist. When a patient was eligible for the study, the study coordinator contacted the research pharmacist to determine the study arm assignment; the research pharmacist then supplied the supplements (or placebo) to the patient. V.W (study biostatistician) generated the random allocation sequence, P.R.C. and other participating urologist investigators at the University of California San Francisco (UCSF) enrolled the participants. All participants, study coordinators, and investigators except V.W. were blinded as to the intervention assignment.

### Study Population and Eligibility Criteria

Detailed inclusion and exclusion criteria were previously reported [13]. Briefly, this study was conducted among men with low burden prostate cancer who met the following criteria: histologically documented prostate adenocarcinoma; extended pattern biopsy within two years of study enrollment with a Gleason sum 6 or lower and no pattern 4 or 5; no more than 33% of biopsy cores positive for cancer; and no more than 50% of the length of a tumor core involved by carcinoma; three serum PSA levels performed at least 2 weeks apart over the past year prior to randomization and all PSA levels < = 10 ng/ml. Target accrual for this study was 97 men. The sample size was based upon the primary study aim using Fisher's exact test with a power of 81% to investigate the difference in proportions of the change in 3 months in specific genes between each supplement and the placebo arm. All men provided verbal and written consent for participation, and this study was approved in April 2003 by the UCSF Human Research Protection Program, which is UCSF's Institutional Review Board.

### Study outcomes

The primary outcome of the trial was limited to change in 2 specific genes (*IGF-1 and COX-2*) and has been reported previously [13]. The current report provides results for the secondary, exploratory aim, that was to investigate the changes in global gene expression and modulation of canonical pathways in normal prostate tissue between baseline and 3 month biopsies, and between diet groups at baseline (see below).

### Biopsy Processing

To procure fresh tissue for gene expression analysis, four 18-gauge core needle core biopsy samples were collected from each participant at baseline and at the three month follow-up (end of intervention).

### RNA Amplification and Microarray Analysis

Details of the RNA amplification and microarray analysis have been previously described [4], [29]. All data are MIAME compliant, and the raw data has been deposited in to GEO under super-series accession #GSE27140.

### Array quality, removal of print run bias and differential expression analysis

To identify differentially expressed genes between treatment arms at baseline, a linear model was fit for each gene expressed as the log2 ratio as the response variable and treatment arm the independent variable, for arm pairs. Diet conditions were dichotomized (high versus low for fish or tomato consumption) to test the relationship between diet with gene expression at baseline. To compare the effect after 3 months between each supplement and the placebo, the difference in the log2 ratios for each gene between the two time points (baseline and month 3) was used as the response variable. The probability values were adjusted by controlling the false discovery rate [30]. A change in gene expression was identified as significant if the false discovery rate was less than 0.05, meaning that fewer than 5% of false findings would be expected among the genes declared to be differentially expressed. The number of tests (multiple comparisons) performed was 39,347, equal to the number of cDNA probes in the microarray. All linear models were fit using the limma package [31], [32] in Bioconductor.

### Canonical Pathway Analysis

A table containing 39,347 cDNA probes and their corresponding Genbank accession ID and expression values (fold change and unadjusted p-value) were uploaded into Ingenuity Pathways Analysis (IPA) software (Ingenuity Systems, Redwood City, CA). IPA was able to map 20,490 Genbank accession IDs to genes (or molecules) present in its database. IPA then assigned gene ontology descriptions of biological function and generated networks of gene interactions on the basis of information retrieved from the software's literature database. Genes that met the cut-off (discussed in the results section) were included in the pathway analysis. Canonical pathways analysis identified the significant pathways from the Ingenuity Pathways Analysis library of canonical pathways that reflected the gene expression in this dataset. Fisher's exact test was used to calculate a probability value that indicated the association between each gene in the dataset and the canonical pathway. A probability value ≤0.05 was chosen to indicate a statistically significant over-representation of molecules in our experiment compared with a curated pathway.

## Results

### Patient characteristics

We enrolled 97 men (target accrual) between October 2003 and December 2007. Baseline clinical and demographic data of the patient cohort were previously described in detail [13]. Thirteen (13) participants were excluded due to ineligibility (n = 5), voluntary withdrawal (n = 3), disease progression (n = 4), or protocol violation (n = 1). The remaining 84 participants comprised the study sample for analysis.

### Tumor tissue yield

Four core biopsies were collected from each of the 84 patients at baseline and at 3 months. Two of the four cores for each patient were obtained from area(s) containing tumor as previously diagnosed and the remaining two were obtained from area(s) with no involvement. Of all the 168 biopsies taken from regions with tumor involvement, only 20% contained tumor, i.e. 35 (20.8%) baseline biopsy cores from 26 patients and 30 (17.9%) 3-month biopsy cores from 23 patients. As expected, the tumor yield was low given initial patient eligibility criteria. Per our prior hypotheses, the gene expression analyses focused on microarray data from morphologically normal prostate tissue samples for all 84 patients.

### Microarray data and quality assessment

A standardized qualitative assessment of array quality was performed using the Bioconductor arrayQuality package [33]. Because a patient may have samples on 2 to 4 arrays at both baseline and 3 months, an array with the best quality measures for each time point was chosen. A total of 84 pairs representing a baseline and three month gene expression data from each patient were subjected to global normalization and differential expression analysis.

Since the cDNA microarrays were printed in 7 batches over 3 years, the effect of print batch was explored. Unsupervised hierarchical clustering revealed that samples hybridized to microarrays from the same print batch clustered together. An attempt to remove the print batch residual (see Methods S1) was not completely successful, although a considerable improvement was observed (Figure S1). Of note, samples did not cluster based on treatment arms or time point (data not shown).

To verify the quality of the microarray data, we compared the baseline gene expression from morphologically normal tissue with available matched tumor tissue from 10 patients. Differential expression analysis revealed genes that have been previously reported to be up regulated in prostate cancer, e.g. HPN [34] and AMACR [35] (data not shown).

To determine comparability of study arms, baseline gene expression profiles for supplementation groups, lycopene (n = 29) and fish oil (n = 27), were each compared to the placebo group (n = 28). Differentially expressed genes were not detected, suggesting that baseline gene expression across all intervention and placebo groups were not significantly different. As reported previously, baseline demographic and clinical characteristics were also similar across treatment arms [13].

### Fish and tomato consumption: baseline gene expression and pathway analysis

Prior to randomization, patients were stratified based on self-reported fish and tomato consumption (i.e., high fish (n = 26), low fish (n = 58); and high tomato (n = 49) and low tomato (n = 35)). Comparison of baseline gene expression profiles between different nutrient groups revealed no differentially expressed genes after adjustment for multiple comparisons.

Because no individual gene met the threshold for statistical significance (adjusted p-value <0.05), we explored potential biological information contained in lists of genes that showed the largest difference between groups being compared [36]. In this study, we chose the unadjusted p-value (not corrected for multiple comparisons) as a means to rank the genes from top to bottom i.e., those at the top are the most differentially expressed genes between two groups. We then used the threshold (unadjusted p-value ≤0.05) to select the top genes to be included in the pathway analysis. For example, applying the threshold to the rank-ordered genes associated with self-reported high dietary consumption of tomato or fish yielded 482 and 192 top genes, respectively (Tables S1 and S2). Each of these lists of genes was then separately used as the input for pathway analysis via IPA. Results of the canonical pathway analysis revealed several interesting pathways that may be associated with high dietary consumption of tomato or fish (Tables 1 and 2). For example, docosahexaenoic acid (DHA) and insulin receptor signaling were modulated among men with high fish consumption versus men who consumed less fish. High tomato consumption revealed the modulation of genes involved in selenoamino acid metabolism. Furthermore, androgen and estrogen metabolism were both modulated among men who routinely ate more fish and tomatoes versus men who consumed less.

**Table 1:** Pathway analysis of gene expression in morphologically normal prostate tissue in men who have high dietary intake of tomato (n = 49) compared with low dietary tomato intake (n = 35).

Ingenuity Canonical Pathways	p-value
Selenoamino Acid Metabolism	0.0029
Hepatic Cholestasis	0.0048
Oxidative Phosphorylation	0.0052
Ubiquinone Biosynthesis	0.0055
Androgen and Estrogen Metabolism	0.0079
Stilbene, Coumarine and Lignin Biosynthesis	0.0098
Methionine Metabolism	0.0105
Methane Metabolism	0.0135
CD27 Signaling in Lymphocytes	0.0339

**Table 2:** Pathway analysis of gene expression in morphologically normal prostate tissue in men who have high dietary intake of fish (n = 26) compared with low dietary fish intake (n = 58).

Ingenuity Canonical Pathways	p-value
Aminoacyl-tRNA Biosynthesis	0.00001
Biosynthesis of Steroids	0.0001
Glycosaminoglycan Degradation	0.0017
Tryptophan Metabolism	0.0028
*Nrf2*-mediated Oxidative Stress Response	0.0069
Sphingolipid Metabolism	0.0079
Galactose Metabolism	0.0081
Pantothenate and CoA Biosynthesis	0.0091
Endoplasmic Reticulum Stress Pathway	0.0191
Inositol Metabolism	0.0219
Docosahexaenoic Acid (DHA) Signaling	0.0219
Hepatic Cholestasis	0.0269
C21-Steroid Hormone Metabolism	0.0275
Butanoate Metabolism	0.0275
Androgen and Estrogen Metabolism	0.0288
Stilbene, Coumarine and Lignin Biosynthesis	0.0302
Glycosphingolipid Biosynthesis - Ganglioseries	0.0372
Insulin Receptor Signaling	0.0380
Caveolar-mediated Endocytosis	0.0389
Sonic Hedgehog Signaling	0.0407
Cell Cycle: G1/S Checkpoint Regulation	0.0417
N-Glycan Biosynthesis	0.0417
N-Glycan Degradation	0.0447

### Lycopene or fish oil supplementation: gene expression and pathway analysis

To investigate the effect of lycopene or fish oil supplementation on the prostate, the change in expression from men who took lycopene or fish oil supplements were compared to men in the placebo group. After correcting for multiple testing, no individual gene met the threshold for statistical significance (adjusted p-value ≤0.05).

Similar to the approach described above, we applied the cut-off (unadjusted p-value ≤0.05) to the rank-ordered genes associated with lycopene or fish oil supplementation. To increase the stringency of the analysis, we only included genes that had ≥1.5 fold change compared to the placebo. Using these thresholds, 57 and 80 genes (Tables S3 and S4) for lycopene and fish oil, respectively, were included for pathway analysis. Results of the canonical pathway analysis revealed the modulation of the arachidonic acid metabolism in the fish oil arm (p = 0.01) compared to placebo; while *Nrf2*-mediated oxidative response was observed in both supplement arms when each was compared to placebo (p = 0.01, fish oil; p = 0.001, lycopene; Table 3 and 4).

**Table 3:** Pathway analysis of gene expression in morphologically normal prostate tissue in men who took lycopene supplements for three months (n = 29) compared with placebo (n = 28).

Ingenuity Canonical Pathways	p-value
*Nrf2*-mediated Oxidative Stress Response	0.0014
Apoptosis Signaling	0.0072
Ceramide Signaling	0.0089
LPS/IL-1 Mediated Inhibition of RXR Function	0.0098
Glutamate Metabolism	0.0178
Axonal Guidance Signaling	0.0380
Glutathione Metabolism	0.0389
PXR/RXR Activation	0.0479

**Table 4:** Pathway analysis of gene expression in morphologically normal prostate tissue in men who took fish oil supplements for three months (n = 27) compared with placebo (n = 28).

Ingenuity Canonical Pathways	p-value
*Nrf2*-mediated Oxidative Stress Response	0.0123
Arachidonic Acid Metabolism	0.0135
Glutathione Metabolism	0.0204
Cyanoamino Acid Metabolism	0.0209
Metabolism of Xenobiotics by Cytochrome P450	0.0316
Alanine and Aspartate Metabolism	0.0324
GABA Receptor Signaling	0.0437
Nitrogen Metabolism	0.0457

## Discussion

The MENS (Molecular Effects of Nutritional Supplements) study was a double-blinded placebo-controlled randomized clinical trial that was developed based on the epidemiological and clinical evidence that linked lycopene and fish oil with reduced prostate cancer incidence and progression [10], [11], [12]. This trial demonstrated the feasibility and safety of studying the effects of nutritional supplements on prostate gene expression in men with low-risk prostate cancer opting for active surveillance. In addition, this study provided further evidence that active surveillance can offer a unique window of opportunity for investigating potential chemopreventive agents [37], [38]. Other trials including the Men's Eating and Living Study (MEAL) also provide important data on the feasibility of implementing clinical trials of dietary intervention in men with low risk prostate cancer [4], [15], [39], [40], [41].

We examined the relationship between baseline gene expression patterns and self-reported dietary intake levels of fish and tomato, and studied the effects of short-term lycopene or fish oil supplementation on the change in prostate gene expression. Taking into account the adjustment for multiple comparisons required in microarray analysis, both analyses yielded no individual gene that was differentially expressed. These results suggest that there were no differences between the groups compared, or that changes in expression were too subtle to be detected given a threshold. Modest alterations in gene expression are difficult to distinguish from noise especially when there is a large number of genes tested, limited samples and high variability between individuals [42]. In addition, potential changes in gene expression may have been dampened by a combination of biological and technical factors including: 1) the dosage and formulation of lycopene and fish oil administered in our study did not have the potency to cause changes in gene expression; 2) the intervention period of three months may have been too short; 3) variability due to diverse participants' diets; 4) detectable changes in gene expression may have occurred in tumors but were not available for analysis; 5) the sample size was relatively small and 6) the noise inherent to the cDNA microarray technology (e.g. print batch effect) may have masked the relatively subtle effects on gene expression.

Although no individual gene was significantly identified with either baseline intake levels or change with lycopene or fish oil supplement, we further explored our prostate gene expression data for potential biological clues by performing pathway analyses of genes (not adjusted for multiple comparisons) that showed the largest differences between groups compared [36]. The unadjusted p-value calculated for each gene was used to order and select top ranking genes for pathway analyses. Canonical pathway analyses (in IPA) revealed candidate pathways that were associated with high dietary intake of tomato or fish at baseline as well as subtle gene expression changes after lycopene and fish oil supplementation. These analyses were done to identify potential genes for future study e.g., RT-PCR of selected genes in candidate pathways modulated by dietary or supplementation of these micronutrients.

We observed that high dietary intake of fish modulated genes involved in metabolic pathways including C21-steroid hormone metabolism and insulin receptor signaling in the normal prostate tissue. To the best of our knowledge, associations between fish intake and steroid hormone metabolism or insulin receptor signaling in the prostate have not been reported. However, the modulation of docosahexaenoic acid (DHA) signaling makes sense as DHA is a major component of fish oil. Studies have shown that DHA inhibits growth of prostate cancer cells [43] and may enhance the efficacy of taxanes, and possibly other drugs, such as COX-2 inhibitors [44], [45]. Moreover, we observed that fish oil supplementation modulated genes involved in arachidonic acid metabolism. *In vivo* and *in vitro* experimental studies have suggested that arachidonic acid, an omega-6 fatty acid, plays a role in the stimulation of proliferation genes that may lead to prostate cancer [46]. Lowering omega-6∶omega-3 fatty acid ratio by increased intake of foods rich in omega-3 fatty acids (such as fish) and perhaps by fish oil supplementation may have inhibitory effects on prostate cancer as demonstrated in cell lines and xenografts [5], [12], [21], [46], [47], [48].

Selenoamino acid metabolism pathway was modulated in men who had high tomato consumption. Selenoamino acids are hypothesized to be responsible for the anti-cancer properties of selenium compounds [49]. For example, seleno-methionine selectively induced growth inhibition and apoptosis in prostate cancer cells but not in normal cells [50], consistent with some of the observational epidemiological data suggesting that selenium may prevent prostate cancer. Of note, selenium supplementation was not associated with reduction in incidence of prostate cancer in the Selenium and Vitamin E Cancer Prevention Trial (SELECT) [51]. We also observed that lycopene supplementation modulated signaling pathways including apoptosis and ceramide signaling. Ceramide is a family of lipid molecules that promote apoptosis and cell cycle arrest [52]. Direct associations between lycopene and ceramide signaling have not been reported, however, lycopene's apoptotic effects have been documented in both xenografts and cancer cell lines [53], [54], [55]. Interestingly, modulation of androgen and estrogen metabolism was observed in men with high consumption of fish and tomatoes. Many dietary factors, including a high omega-3 fatty acid diet, have been documented to impact androgen or estrogen levels [56].

The *Nrf2*-mediated oxidative stress response pathway was observed to be modulated by both lycopene and fish oil supplement vs. placebo. Studies in mice have shown that the loss of *Nrf2* function correlated with increased reactive oxygen species and DNA damage leading to the transformation of normal prostate tissue [57]. In addition, a study in knock out mice revealed a link between the chemopreventive effects of soy isoflavones and the role of *Nrf2* in modulating signaling pathways involved in the prevention of prostate cancer [58]. Recent studies have shown that other food-based or pharmacological compounds also exert their chemopreventive effects via the *Nfr2* signaling pathway by mediating increased activity of cytoprotective enzymes, e.g. phase II detoxifying and antioxidant enzymes [59], [60], [61], [62]. Taken together, the modulation of the *Nrf2* signaling pathway may be an important molecular mechanism involved in chemoprevention by several agents including lycopene and fish oil. Interventions that target the *Nrf2* pathway may offer a promising strategy for chemoprevention.

Exploring the top genes (most differentially expressed) selected after applying specified cut-offs revealed intriguing results. For example, *KLK3* (aka prostate specific antigen or PSA) was up regulated in the lycopene arm (6.2 fold change) compared to the placebo group (Table S3) while *KLK3* was down regulated (−2.5 fold change) in men who had high fish consumption at baseline compared to men who consumed less fish (Table S2). Of note, *IGF1* was down regulated in the fish oil arm (−1.5 fold change) compared to the placebo group (Table S4). Down regulation of *IGF1* was not observed in the lycopene arm. While provocative, care should be exercised when interpreting these results since these genes were not significant after adjustment for multiple comparisons.

Except for *Nrf2*-mediated oxidative stress response, none of the pathways identified after supplementation overlapped with the pathways identified at baseline. Interestingly, there are on-going debates [63] whether obtaining nutrients from whole food has the same health impacts as supplementation. Besides lycopene and fish oil (DHA/EPA), other naturally occurring nutrients in whole tomatoes (e.g. other carotenoids) [64] and fish (e.g. alpha-linolenic acid) may also be important to the positive health effects of these foods. However, interpretations made from comparing the results between the analysis of baseline and supplementation groups in this study should be done with caution because the baseline and intervention analyses were originally designed to measure different outcomes.

Recent research in personalized nutrition has demonstrated that nutrients may interact with an individual's genotypic and phenotypic background [65], [66]. For example, supplements may affect gene expression in different directions (i.e. up- or down regulation) depending on the genotype/phenotype of the individual. Furthermore, nutrients may produce different phenotypes in patients with different genotypes. A P450 cytochrome allele, for instance, may metabolize a dietary substrate to a bioactive form, in contrast to another allele that produces an inactive metabolite [67]. Hence, variability among patients may cancel out the effects of the dietary factors if analyzed at the global level resulting in an undetectable net change. As more examples of diet-gene interactions are discovered, increased power and sophistication of clinical trials will become possible.

A more recent report from the Selenium and Vitamin E Cancer Prevention Trial (SELECT) demonstrated the cell and tissue specific effects of selenium and vitamin E on gene and protein expression in the prostate [68]. They detected differentially expressed genes (relative to the placebo group) in men who were taking selenium or vitamin E or in combination, only when cell type (normal epithelium, stroma or tumor) was taken into consideration. In the MENS study, morphologically normal tissue used for gene expression contained both stroma and normal epithelium; hence, cell type specific analysis was not possible.

Although differential expression analysis did not detect significant individual genes, our exploratory analysis revealed candidate *in vivo* pathways that may be modulated by dietary fish and tomato intakes or by lycopene and fish oil supplementation. Our study provides a platform to investigate the bioactivity and relevance of nutrients in prostate cancer. Understanding molecular mechanisms by which micronutrients affect gene expression would have a great impact on the development of prevention and treatment strategies in prostate cancer, especially for men electing active surveillance. Finally, improvements in commercial RNA amplification, oligonucleotide microarray platforms, potency and longer intervention periods are important factors to consider when performing gene expression studies for agents that have subtle effects on the human transcriptome.

## Supporting Information

Figure S1 Samples were hybridized to 7 different batches of printed cDNA microarrays (#11, 12, 14, 17, 18, 19 and 20). Print run effect was controlled by fitting a linear model with log2 ratio as response and batch effect as explanatory variable and using the residuals from the fit for further analyses.
(DOC)

Table S1 Differentially expressed genes (unadjusted p-value ≤0.05) in morphologically normal prostate from men with high dietary intake of tomato (n = 49) compared with low dietary intake (n = 35).
(XLS)

Table S2 Differentially expressed genes (unadjusted p-value ≤0.05) in morphologically normal prostate from men with high dietary intake of fish (n = 26) compared with low dietary intake (n = 58).
(XLS)

Table S3 Differentially expressed genes (unadjusted p-value ≤0.05, >1.5 fold change) in morpholigically normal prostate between men randomized to the lycopene (n = 29) and placebo arms (n = 28).
(XLS)

Table S4 Differentially expressed genes (unadjusted p-value ≤0.05, >1.5 fold change) in morpholigically normal prostate between men randomized to the fish oil (n = 27) and placebo arms (n = 28).
(XLS)

Methods S1 (DOC)

Checklist S1 (DOC)

Protocol S1 (PDF)
