# Supplementary material for: Gene Expression and Biological Pathways in Tissue of Men with Prostate Cancer in a Randomized Clinical Trial of Lycopene and Fish Oil Supplementation
Source: PLoS One. 2011 Sep 1;6(9):e24004. doi: 10.1371/journal.pone.0024004 (PMC3164676; doi:10.1371/journal.pone.0024004)
Supplement: Figure S1 — Samples were hybridized to 7 different batches of printed cDNA microarrays (#11, 12, 14, 17, 18, 19 and 20). Print run effect was controlled by fitting a linear model with log2 ratio as response and batch effect as explanatory variable and using the residuals from the fit for further analyses. (DOC) [file pone.0024004.s001.doc]

Before print run bias removal

After print run bias removal

Figure S1. Samples were hybridized to 7 different batches of printed cDNA microarrays (#11, 12, 14, 17, 18, 19 and 20). Print run effect was controlled by fitting a linear model with log2 ratio as response and batch effect as explanatory variable and using the residuals from the fit for further analyses.
