# Supplementary material for: Gene Expression and Biological Pathways in Tissue of Men with Prostate Cancer in a Randomized Clinical Trial of Lycopene and Fish Oil Supplementation
Source: PLoS One. 2011 Sep 1;6(9):e24004. doi: 10.1371/journal.pone.0024004 (PMC3164676; doi:10.1371/journal.pone.0024004)
Supplement: Methods S1 — (DOC) [file pone.0024004.s006.doc]

**SUPPLEMENTARY METHODS**

**Tissue collection.** Cores were obtained from the peripheral zone under ultrasound guidance. Based on previous biopsy results, two cores were obtained from areas containing tumor and two cores from areas with no involvement. Core samples were immediately embedded in OCT medium and flash-frozen over dry ice. Using a cryostat, 17 sections (14 µM each) were cut from each tissue block and the 8^th^ section was processed for hematoxylin-eosin (H&E) staining. The H&E slides were reviewed for each case by the study pathologist (JS) to confirm grade/extent of disease as well as to identify normal and tumor regions for gene expression analyses. For some cases, the 17^th^ section was used to verify tumor diagnosis via immunohistochemistry to confirm loss of high molecular weight cytokeratins using DAKO #M0630 [1]. The remaining tissue sections were microdissected using the H&E slide as a guide for tumor and normal tissue areas. RNA was isolated from microdissected tissue using RNEasy columns (Qiagen, Valencia, CA).

**RNA amplification and microarray experiments.** 25-100 ng of total RNA, side by side with an equal quantity of universal human reference RNA (Stratagene #740000), was linearly amplified through two rounds of modified *in vitro* transcription [2]. Amplified RNAs were converted to aminoallyl-modified cDNA, coupled to *N-*hydroxysuccinimidyl esters of Cy3 or Cy5 (Amersham Biosciences, Piscataway, NJ)[3], and hybridized to arrays at 63° C for 12-16 h [4]. Slides were then washed and immediately scanned and analyzed with Axon Imager 4000b (Axon Instruments, Foster City, CA), using GENEPIXPRO3.0. Gene expression values were expressed as log2 ratios representing the relative abundance of test RNA compared with reference RNA derived from the normalized intensities.

The microarray was comprised of 39,347 cDNAs representing 20,862 annotated genes. Clone annotation was updated using Unigene Build #208 of the human genome accessed through the SOURCE database [5], and UCSC genome database and software tools [6] for clones without Unigene annotation. Clones with DNA sequence corresponding to repetitive elements, or which represented non-transcribed DNA were removed from the final dataset.

**Array quality, normalization, and removal of print run bias.** All analyses were done using the freely available R/Bioconductor software [7,8]. Array quality was analyzed using the arrayQuality package in Bioconductor. Each array was normalized using the print tip loess method, with data from different batches combined by mapping using gene symbol and averaging probe values from replicate genes. The print run effect was then controlled by fitting a linear model with the response expressed as the log2 ratio and batch effect as the explanatory variable and using the residuals from the fit for further analyses.

**REFERENCES**

1. Wojno KJ, Epstein JI (1995) The utility of basal cell-specific anti-cytokeratin antibody (34 beta E12) in the diagnosis of prostate cancer. A review of 228 cases. Am J Surg Pathol 19: 251-260.

2. Baugh LR, Hill AA, Brown EL, Hunter CP (2001) Quantitative analysis of mRNA amplification by in vitro transcription. Nucleic Acids Res 29: E29.

3. Hughes TR, Mao M, Jones AR, Burchard J, Marton MJ, et al. (2001) Expression profiling using microarrays fabricated by an ink-jet oligonucleotide synthesizer. Nat Biotechnol 19: 342-347.

4. DeRisi J, Penland L, Brown PO, Bittner ML, Meltzer PS, et al. (1996) Use of a cDNA microarray to analyse gene expression patterns in human cancer. Nat Genet 14: 457-460.

5. Diehn M, Sherlock G, Binkley G, Jin H, Matese JC, et al. (2003) SOURCE: a unified genomic resource of functional annotations, ontologies, and gene expression data. Nucleic Acids Res 31: 219-223.

6. Karolchik D, Kuhn RM, Baertsch R, Barber GP, Clawson H, et al. (2008) The UCSC Genome Browser Database: 2008 update. Nucleic Acids Res 36: D773-779.

7. Gentleman R, Carey V, Dudoit S, Irizarry R, Huber R (2005) Bioinformatics and Computational Biology Solutions using R and Bioconductor. New York: Springer.

8. R Development Core Team (2005) R: A language and environment for statistical computing. R Foundation for Statistical Computing. Vienna, Austria.
