# Supplementary material for: Gene Expression and Biological Pathways in Tissue of Men with Prostate Cancer in a Randomized Clinical Trial of Lycopene and Fish Oil Supplementation
Source: PLoS One. 2011 Sep 1;6(9):e24004. doi: 10.1371/journal.pone.0024004 (PMC3164676; doi:10.1371/journal.pone.0024004)
Supplement: Protocol S1 — (PDF) [file pone.0024004.s008.pdf]

UCSF Protocol #: 03553

TITLE: The Molecular Effects of Nutrition Supplements (MENS) Prostate Study

Coordinating Center: University of California, San Francisco Comprehensive Cancer Center

Principal Investigator: Peter R. Carroll, MD
Professor and Chairman, Department of Urology
UCSF Comprehensive Cancer Center
1600 Divisadero St.; 3rd floor
San Francisco, CA 94115
ph: (415) 353-7098
fax: (415) 353-7093
pcarroll@urology.ucsf.edu

Co-Investigator: Christopher M. Haqq, M.D., Ph.D.

Sub-Investigators
June Chan, Sc.D.
Matthew Cooperberg, M.D., M.P.H.
Katsuto Shinohara, M.D.
Christopher Kane, M.D.
Maxwell Meng, M.D.
Kirsten Greene, M.D.

Statistician: Vivian Weinberg, PhD
Principal Biostatistician
UCSF Comprehensive Cancer Center
513 Parnassus Ave., Box 0127
San Francisco, CA 94143
Ph: (415) 502-5472
Fax: (415) 476-0624
weinberg@cc.ucsf.edu

Original Version: December 4, 2002
Amendment #16: April 16, 2007

Responsible Data Manager: Sarah Dumican, M.S., R.D.
(415) 885-3673

Investigational Agents: Lycopene
Fish Oil

1. Objectives 4

2. Background 5
 2.1 Scientific background 5
 2.2 Diet and prostate cancer 5
 2.3 Lycopene/tomato products 6
 2.4 Fish oil/omega-3 fatty acid 9
 2.5 Clinical backgroud: watchful waiting in prostate cancer 11

3. On-study guidelines, study design 14
 3.1 General study design 14
 3.2 Outcome measures 14

4. Subjects 16
 4.1 Who and why 16
 4.2 Eligibility 17
 4.3 Inclusion of women and minorities 19
 4.4 Total number 19
 4.5 Recruitment 19
 4.6 Method of treatment assignment and blinding 19

5. Treatment Plan 20
 5.1 Table of drug administration 20
 5.2 Written description of drug dosing 20
 5.3 Duration of therapy 21
 5.4 Other pertinent info about administration 21

6. Agent Formulation and Procurement 21
 6.1 Lycopene 22
 6.2 Fish oil 23

7. Dose Modifications 24

8. Study Assessments 25
 8.1 Registration 25
 8.2 Screening assessments 25
 8.3 Assessment during the treatment period 28
 8.4 Early termination or final visit 29
 8.5 Additional safety follow-up 29
 8.6 Study table: timing of PE, labs, restaging 29

9. Criteria for Response, Progression, and Relapse 32
 9.1 Definitions 32
 9.2 Guidelines for evaluation 32
 9.3 Molecular 32
 9.4 Clinical 32

10. Safety and ethical considerations 33
 10.1 Safety plan 33
 10.2 Assessment of safety 33
 10.3 Adverse event reporting 36
 10.4 Consent 36
 10.5 Confidentiality, risks, and protection of subjects 37
 10.6 Ethics 38
 10.7 Compliance with laws and regulations 38

11. Ancillary Therapy 39
11.1 What is allowed during treatment 39
12. Removal of Patients from Protocol Therapy 39
13. Statistical Considerations 39
14. Qualifications of investigators 42
15. References 43

1. Objectives

1.1 Primary: To measure gene expression in normal prostate tissue acquired by needle biopsy in men assigned to placebo, lycopene or fish oil nutritional supplements at baseline and after intervention. The primary outcome measure is a two-fold decrease in mRNA transcript level. The primary study endpoint is the difference in the proportion of the patients in a nutritional supplement arm compared to a placebo control arm achieving a two-fold decrease in the gene of interest. Quantitative polymerase chain reaction will assess modulation of expression of the *insulin-like growth factor* 1 (*IGF1*) and the cyclooxygenase 2 (*Cox-2)* gene in lycopene and fish oil recipients respectively.

1.2 Secondary: We will use cDNA microarrays with 30,000 independent clones to identify new candidate molecular targets for lycopene and omega-3 response pathways deserving further study.

1.3 Secondary: To correlate baseline gene expression patterns determined by cDNA array analysis with self-reported dietary intake. Based on previous epidemiologic, *in vitro*, and *in vivo* studies, we hypothesize that higher intake of total energy, fat/meat/animal products, dairy/calcium, and lower intakes of fish, vegetables, tomatoes/lycopene, vitamin E, and selenium will be associated with particular gene expression profiles.

1.4 Secondary: We will assess if there are gene expression patterns correlated with progression or lack of progression as assessed at a time point 12 months after study entry.

1.5 Secondary: We will assess if lycopene or omega-3 supplements affect the incidence of tumor progression.

2. Background

2.1 Scientific background

Prostate-specific antigen (PSA) screening has resulted in dramatic improvements in early detection and treatment of prostate cancer. However, the natural history of the disease may be protracted, and all available treatments potentially exert a significant negative impact on patient quality of life. In the Molecular Effects of Nutrition Supplements (MENS) trial, men with favorable-risk prostate cancer, who opt initially for watchful waiting, will be offered participation in a prospective randomized placebo- controlled 3-month nutritional intervention study with a genetic correlative science outcome, using supplementation with lycopene or fish oil as a possible means to modulate prostate cancer progression. Research needle biopsies will be taken at the beginning of the study and after intervention. The primary outcome measure is a two-fold up or two-fold down change in gene transcript level between three-month and baseline biopsies in normal prostate tissue. In particular, we hypothesize that lycopene supplementation will be associated with decreased expression of the *insulin-like growth factor* (*IGF*) gene, and that fish oil supplementation will be associated with down-regulation of the *Cox-2* gene at 3 months. Furthermore, we will identify new candidate molecular targets for lycopene and omega-3 response pathways deserving further study since microarrays will be used to assess an additional 30,000 genes for each study biopsy.

2.2 Diet and prostate cancer

Several food and nutrients have been studied in relation to prostate cancer. In particular, lycopene/tomato products, fish, vitamin E, selenium, total and specific vegetables, and soy/legumes have been routinely inversely associated with risk of prostate cancer in observational studies or clinical trials. Vitamin E and selenium are currently being examined in a large clinical trial for the primary prevention of prostate cancer (SELECT trial). While total and specific vegetables and soy/legumes have been associated with a reduced risk of prostate

cancer incidence, food-based interventions cannot be blinded and "greater vegetable intake" represents a multitude of nutrients and phyto-chemicals that may influence and interact with many genetic pathways. Attempting to study many nutrients and pathways simultaneously can inhibit the ability to identify any one single pathway. Thus, we chose to focus our study on elucidating the molecular effects of lycopene and fish oil in a randomized, double-blinded, placebo-controlled clinical trial among men with prostate cancer. We will stratify patients with regards to baseline intake of fish and tomato products before randomization, to ensure that men in both experimental arms are a balanced sample of the study population. Below, we outline the evidence supporting the beneficial effects of lycopene and fish oil. We conclude this section with an overview of other dietary risk factors for prostate cancer that we will investigate in an exploratory fashion by comparing the self-reported diet records and questionnaire data with different molecular and genetic markers/indices.

2.3 Lycopene/tomato products

General

Lycopene, most commonly found in tomatoes, is the most efficient quencher of singlet oxygen among the common carotenoids [1] and is the predominant carotenoid in plasma [2] [3] and various tissues [4], including the prostate gland [5]. The Health Professionals Follow-up Study found that high lycopene intake was correlated with a 21 percent lower risk of prostate cancer; and tomato sauce, the major predictor of serum lycopene levels, was the strongest dietary predictor of reduced prostate cancer risk (36% reduction for high versus low intake) [6] This relationship was independent of other factors and stronger for advanced cases. Two studies of pre-diagnostic serum carotenoids and prostate cancer observed inverse associations for lycopene. The Physicians Health Study found that being in the fifth vs. the first quintile of plasma lycopene levels was associated with an approximate 25% reduction in overall prostate cancer risk, and a 44% statistically significant reduction in risk of aggressive cancer [7]. Lycopene levels in

African American men are lower than in Caucasian men, paralleling the difference in prostate cancer incidence seen in these two groups[8].

Is the lycopene itself protective, or is it merely a marker for some other diet or behavioral trait? Recently, investigators reported statistically significant reductions in levels of DNA oxidative damage and PSA before and after intervention with a tomato sauce-based (lycopene-rich) diet given three weeks prior to prostatectomy among 30 prostate cancer patients [9]. In addition, one recent study compared 15 men taking 30mg lycopene supplement for three weeks with 11 men given no intervention, who then underwent radical prostatectomy [10]. In this study, both decreased tumor volume and decreased incidence of high grade prostate intraepithelial neoplasia (PIN) were found. A Southwest Oncology Group Trial is planned to confirm these findings. In addition, a case report details the benefit of lycopene in a patient referred to hospice care after failure of cytotoxic chemotherapy. The patient reported taking both lycopene supplements and saw palmetto as alternative medicines and achieved a lasting complete remission of his advanced prostate cancer[11]. Given these findings, which show that lycopene itself has protective effects, we hypothesize that lycopene will have a distinct gene expression signature in prostate tissue.

The molecular mechanism of lycopene action on target cells is unknown, and may extend beyond quenching oxygen free radicals. Breakdown of any carotenoid, including lycopene, results in the generation of vitamin A and other retinoids. Recently, prostate short chain dehydrogenase/reductase was found to be specifically a retinal reductase, indicating that prostate tissue may have a unique metabolism of retinoid molecules[12]. Furthermore, lycopene[13], or acyclo-retinoic acid, one of the products of lycopene metabolism, was shown to cause prostate cells to undergo apoptosis *in vitro*[14]. Thus, the molecular mechanism of lycopene action may include induction of the apoptotic cascade. In addition, lycopene supplementation was associated with a decrease in serum levels of insulin-like growth factor I (IGF-1) in two recent small trials[15, 16]. Serum IGF-I has been linked to greater risk of prostate cancer in several studies, including two conducted by our group (Chan JM et al, Science 1998, Chan JM et al,

JNCI, 2002). Since IGF modulates the protein kinase B/Akt kinase pathway critical to prostate cell survival[17, 18], a reduction in IGF levels would be expected to promote apoptosis in prostate cancer. The cDNA arrays we will use in our study (see Sections C and D, preliminary data and research methods), include sequences encoding the prostate short chain reductase gene, the retinoic acid receptors, apoptotic mediators and components of the IGF signaling pathway, to maximize the chance to detect the *in vivo* mechanism of lycopene action on the prostate.

Pharmacokinetics

Lycopene, a non provitamin A carotenoid, can act directly as an antioxidant[19]. At lycopene levels readily achieved in humans, glutathione transferase and superoxide dismutase activities are induced in animal models[20]. Small differences in bioavailability are seen depending on which type of oil emulsion is used as the vehicle in lycopene administration[21], and lycopene bioavailability is greatly enhanced by food processing, usually by making a tomato sauce[22].

Toxicology

The toxicity of lycopene was determined in a thirteen week study of male and female Wistar rats fed several very large doses – 0, 500, 1500 and 3000 mg/kg[23]. No statistically significant, dose-related effects on body weight, body weight gain, food consumption, hematology, urinalysis, clinical chemistry or ophthalmoscopic parameters were seen in any of the lycopene product or lycopene formulation matrix groups in comparison to the vehicle control group. No deaths attributed to the test articles occurred during the study and the only clinical finding and at necropsy was the presence of red pigment in the feces and gastrointestinal tract that was associated with the red-pigmented test materials. Thus, there are no significant toxic findings even when lycopene is administered at very high dose levels[23]. Organ histology at the time of animal sacrifice was unchanged relative to controls.

Phase I trials

Recently a small clinical study of 15 men assigned supplement and 11 controls showed a decrease in PSA, decrease in tumor volume and decrease in IGF-1 levels after 3 month consumption prior to radical prostatectomy surgery[16]. While this study is unique in that the population considered had prostate cancer, the many additional studies of lycopene consumption addressing cardiovascular, ocular or other clinical endpoints have not been complicated by any adverse effects[24-31]. Together these studies represent treatment of hundreds of subjects without ill effect.

2.4 Fish oil/omega-3 fatty acid

General

There is provocative evidence for a potential protective effect of fish intake on prostate cancer risk from a large U.S. cohort study [32], a Swedish case-control study [33], a smaller New Zealand case-control study[34], cross-sectional serum studies[35], and several experimental studies in prostate cancer cell lines[36]. DU-145 xenografts were growth inhibited in nude mice fed a diet rich in omega-3 fatty acids[37]. Furthermore, we have preliminary evidence from the Health Professionals Follow-up Study of a moderate to strong inverse association for post-diagnostic fatty fish intake and risk of prostate cancer recurrence/mortality (unpublished data, *JM Chan*). While this cohort was not specifically designed to study prostate cancer recurrence and mortality and has limited power to detect associations, we (Chan and colleagues at Harvard University) examined post-diagnostic diet relevant to pre-diagnostic diet and risk of prostate cancer recurrence and mortality among 1,584 incident prostate cancer cases, using prospectively collected questionnaire data. In a preliminary analysis, a positive change in intake of fish after diagnosis (e.g. consuming more fish after diagnosis relative to before diagnosis) was strongly protective for risk of prostate cancer recurrence (RR=0.35 for increasing intake by one serving/day, p = 0.0002), independent of other food groups, PSA level at diagnosis, Gleason score of the tumor, stage, and primary treatment choice. Furthermore, the risk of dying from

prostate cancer was inversely associated with consuming more fish, grains, tomato products, and vegetables after diagnosis relative to prior to diagnosis, independent of baseline clinical characteristics.

Pharmacokinetics

Omega-6 fatty acid, also known as arachidonic acid, is metabolized by the COX-2 enzyme to become prostaglandin E2 (PGE2), a molecule implicated in carcinogenesis in part due to its ability to stimulate prostate cell growth *in vitro*[38, 39]. Ratios of omega-3 to omega-6 fatty acid are thought to modulate COX-2 expression at both RNA and protein levels.

Toxicology

The acute and chronic toxicity of dietary fish oil was determined in a twelve-month study of Sprague-Dawley rats fed chow equivalent to human consumption of nine grams fish oil daily. Expected decreases in serum triglyceride, total cholesterol and LDL levels were seen[40]. There were no acute or chronic differences in external appearance, level of activity, daily food consumption, blood cell counts, renal function, thyroid function, prothrombin time or activated partial thromboplastin time, and organ histology at the time of animal sacrifice was unchanged relative to controls.

Phase I trials

Recently a small group of men were studied after three-month intervention comprised of low fat diet with fish oil supplementation. At the three-month assessment of these nine men, the ratio of omega3 to omega6 fatty acids in gluteal fat was increased, and COX-2 expression quantitated by PCR from prostate needle biopsies was decreased in four out of seven patients assessed[41]. While preliminary, these results indicate that fish oil rich in omega-3 fatty acids may influence prostate cancer progression. In addition, no ill effects were found in many additional clinical trials of a similar patient population when fish oil effects on atherosclerosis, or

other clinical endpoints were assessed[42-45]. The cited trials include studies in infants and pregnant women, which highlights the safety of treatment with fish oil as a nutritional supplement.

2.5 Clinical background: watchful waiting in prostate cancer

Prostate cancer is the most common malignancy in men, with 221,000 new cases and 29,000 deaths expected in 2003[46]. Worldwide, incidence of prostate cancer diagnosis is increasing at 3% per year[47]. The advent of prostate specific antigen (PSA) screening has resulted in improvements in early diagnosis of prostate cancer. However, the natural history of the disease may be protracted and only 25-33% of men diagnosed with prostate cancer actually die of the disease[48, 49]. Moreover, available treatments all have a significant negative effect on quality of life[50]. Analysis of tumor registries in the United States[51] and Sweden[52] with fifteen year median follow-up shows that some patients enjoy prolonged survival without definitive treatment[51]. Low grade tumors, in particular, were associated with low 4-30% risk of death from prostate cancer. However, for 876 men with aggressive Gleason pathologic scores of 8 to 10, surgery or radiation provided better five-year overall rates than expectant management. In this US tumor registry study, the ten year prostate cancer specific survival for surgery was 67%, for radiotherapy 53%, and for expectant management 45% [53]. Since PSA screening has led to stage migration with patients often diagnosed with the earliest clinical stage T1c, interest has grown in offering **watchful waiting** – careful surveillance with frequent PSA testing followed by repeat biopsy and referral for definitive treatment when indicated – as an initial treatment recommendation.

A recent analysis of 4,458 men in the CaPSURE longitudinal, observational prostate cancer patient database found that 8.2% of patients, principally those who were older and had favorable risk characteristics, opted for watchful waiting; 52% of these patients underwent secondary treatment within five years, especially those who were younger or had higher PSA scores at diagnosis[54]. A single-institution study of 199 patients, again comprised of older

patients with low-risk tumors, showed that 44% of men underwent prostate cancer treatment by five years, with a disease-specific survival rate of 98% at seven years[55]. There is growing evidence that patients who choose watchful waiting do not experience adverse psychological impact, even when assessed with 10 year median follow-up[56].

To date, most patients opting for watchful waiting have been older, with more comorbidity, or strongly averse to extant treatment alternatives. Initial watchful waiting trials reported on study populations with high clinical T stage, PSA and Gleason scores, and found that several men developed metastasis during observation[57]. However, with the development of better monitoring algorithms, new trials are exploring the possibility of offering safe initial observation to greater numbers of patients. Those who have low-risk tumor characteristics could pursue a trial of initial active monitoring at the time of diagnosis, without sacrificing curative intent, nor exposing the patients to undue risk of disease progression. Such an approach could preserve quality of life and achieve significant cost savings without impairing cure rates. The ongoing Prostate Cancer Intervention Versus Observation Trial (PIVOT) is examining the utility of initial observation in a randomized trial[58], with results expected in approximately five years.

Recently, investigators at Johns Hopkins University and University of Toronto have sought to define a low risk population of patients using the standard staging tools of PSA, biopsy Gleason score, and ultrasound stage. For example, Sunnybrook Hospital in Toronto reported on a cohort of 206 low risk patients with median follow-up of 29 months [59]. Low risk men were defined as stage T1b-T2b, N0, M0, with PSA < 15 and Gleason score <=7. Patients were evaluated every three months for the initial 24 months, and thereafter every six months. Lab testing for PSA, prostatic acid phosphatase (PAP), and serum creatinine was performed at each visit, and patients received a digital rectal exam. Transrectal ultrasound was performed every six months, and repeat transrectal 18 gauge needle biopsy was performed 12 to 18 months after study enrollment. Patients with progressive disease were counseled to stop the trial and undergo definitive surgical or radiation therapy. Progressive disease was defined as either clinical (symptoms of urethral obstruction requiring transurethral resection of the prostate, or evidence –

radiological or clinical - of metastasis), histologic (Gleason score >=8 on the repeat biopsy), or biochemical (the three simultaneous conditions of PSA doubling time < 2 years, final PSA > 8 and $p < 0.05$ on regression analysis of the time derivative of lnPSA). In this study 137/206 patients remained on study without evidence of disease progression. Of the 69 patients who discontinued the study, only 36 (17%) had evidence of disease progression – 15 had clinical progression (5 underwent transurethral prostatic resection, and ten had an enlarging nodule on DRE), 16 had PSA progression and five had histologic progression. Of the remaining patients, 23 voluntarily withdrew, six were withdrawn for protocol violations, and four died of other causes. No patient had evidence of metastasis.

A second trial reported 23 month median follow-up of 98 men with inclusion criteria of Gleason score <=6, less than three of six biopsies containing cancer, no more than 50% involvement of any biopsy, and PSA density < 0.15 ng/ml[60]. Patients were followed at yearly intervals with %free PSA and digital rectal exam. At two years, transrectal ultrasound was performed with 12-core diagnostic needle biopsy. Definitive treatment was recommended if Gleason pattern 8-10 histology was seen, > 2 biopsies were positive for cancer, or if there was > 50% involvement of any biopsy with cancer. Twenty-five of the 81 men (31%) had disease progression at two years. Of these men, 13 underwent radical prostatectomy and 12 of these men had potentially curable cancers (>70% survival at 10 years expected based on pathologic features). The higher rate of progression seen in this trial was most likely an artifact of requiring only sextant biopsies, which allowed higher grade disease to be missed more often at the time of initial biopsy[61]. There was no difference in initial PSA, percent free PSA, PSA density or PSA velocity comparing patients with progression on re-biopsy to those without progression.

These data support the safety and feasibility of our proposed prostate cancer intervention trial. The articles cited demonstrate that definitive treatment for prostate cancer is possible at the first sign of progressive disease, and that with appropriate entry criteria, no man will be denied the opportunity for treatment with curative intent by participating in our study

3. On-study guidelines, study design

3.1 General study design

We propose a randomized placebo-controlled trial of lycopene and fish-oil supplementation among men with newly-diagnosed, favorable-risk prostate cancer who opt for watchful waiting as their primary treatment strategy. Our main outcome will be changes in gene expression in prostate tissue biopsies taken before and after the three-month intervention period. Men will provide dietary surveys and records prior to each biopsy (i.e. before and after the three-month intervention). Serum levels of lycopene and omega-3 fatty acid will be measured before and after the intervention to assess compliance and to conduct correlative analyses.

3.2 Outcome measures

3.2.1 Primary outcome measures

The twofold decrease in mRNA transcript level detected by Taqman quantitative polymerase chain reaction comparing baseline to three-month normal prostate biopsies will be the primary outcome measure. Histologic examination of the needle biopsy tissue will be used to verify that RNA is prepared only from normal prostate areas. (Where possible, areas of tumor histology will be assessed in parallel, though assays based on these tissues will not form part of the primary outcome measure). We will specifically assess the effect of lycopene supplementation on *IGF-1* and the effect of fish oil supplementation on *Cox-2* when compared to the placebo control group.

3.2.2 Secondary efficacy outcome measures

3.2.2.1 The secondary aim of identifying genes correlated with nutritional supplementation with lycopene or fish oil will be accomplished by analyzing gene expression of 30,000 human genes using a cDNA array. Identification of a gene cluster associated with lycopene or fish oil but not placebo will be

accomplished using hierarchical clustering[62], or alternate algorithms that use permutation t-test statistics to account for the multiplicity of tests accomplished using such large scale microarrays[63]. If sufficient nucleic acid samples remain after primary outcome measure assessment, and microarray analysis, confirmatory Taqman quantitative polymerase chain reactions will be used to confirm the specific induction of genes identified in these studies by lycopene or fish oil.

3.2.2.2 To accomplish the secondary aim of identifying genes correlated with baseline dietary intake, patients will be stratified for high and low intake of total energy, fat/meat/animal products, dairy/calcium, fish, vegetables, tomatoes/lycopene, vitamin E, and selenium as defined by the Willett Semi-quantitative Food Questionnaire. Separate microarray data analyses will be performed for each dietary factor by placing all patients with low intake (<= median) into the first half of the data table, and subsequently all patients with high intake (> median). The same standard microarray hierarchical clustering and permutation t-test approaches as in 3.2.2.1 will then be used to identify genes whose expression differs between these groups.

3.2.2.3 We acknowledge that the study is underpowered to assess effects of nutrition supplementation on progression rates, given the heterogeneous nature of prostate adenocarcinoma. Nevertheless, it is compelling to perform exploratory data analysis to examine whether exploratory analysis may detect genes correlated with progression or lack of progression at 12 months in the MENS study. If any correlates are detected, they will be used to formulate a hypothesis for testing in further large scale studies, such as the multi-institutional CALBG or SWOG watchful waiting trials.

3.2.2.4 With watchful waiting, approximately 25% of patients will experience disease progression when assessed 12 months after study entry. To assess whether the

intervention arms are different in outcome, an analysis for efficacy will be performed after 10 patients have been accrued to each of the 3 treatments and followed for 12 months from study entry. If more than 5 of the 10 patients on either supplement arm experience disease progression at twelve months, the study will be closed to further accrual. It is anticipated that this analysis will occur 15 months after the study is opened when approximately 45 patients have been entered on study. A statistician in the UCSF Comprehensive Cancer Center not associated with the design or final analysis will conduct the interim analysis. Similar analysis will be performed when the trial has reached its total accrual goal.

Safety outcome measures

As the agents proposed for study here are widely used without report of adverse effects, and since in clinical studies reported to date there were no adverse effects noted, we do not anticipate that lycopene or fish oil supplementation will adversely affect prostate cancer progression.

4 Subjects

4.1 Who and why

As described in the Background section (2.5) above, many men with favorable-risk prostate cancer may expect extended survival without definitive therapy. There is a need for interventions in these patients that may delay or prevent disease progression with a minimal impact on health-related quality of life (HRQOL). A large proportion of prostate cancer patients use nutritional supplements and other “complementary” treatments toward this end, but few of these treatments have been formally evaluated. A growing number of studies confirm that low-risk clinical and pathologic risk characteristics consistently predict tumors with relatively low short-term metastatic potential, and that close monitoring can identify early those patients opting for deferred intervention who show signs of a higher progression risk. These patients are the target cohort for our study. Patients at risk for prostate cancer seen in the Urologic Oncology Program at the University of California at San Francisco at UCSF/Mt. Zion are potential subjects for this study.

After the 3-month intervention, patients will return to the care of their referring UCSF urologist for subsequent 21 months of follow-up in the MENS study, following which the standard of care is for follow-up at intervals no greater than every six months.

4.2 Eligibility

Inclusion criteria

- Histologically documented, untreated prostate adenocarcinoma, (small cell acinar type).
- Patients must have had an extended pattern biopsy (defined as 8+ cores) within 2 years of the screening date. Potential subjects who meet all of the eligibility criteria EXCEPT for the aforementioned extended pattern biopsy within two years of the screening date, may enroll in the study if they have an extended pattern clinical biopsy scheduled within 6 weeks of the Intervention start date. They must also be willing to have an additional 4 biopsy cores taken for research at this appointment, as well as have the other baseline data collected (QOL, FFQ, 3-day food diary, blood and urine specimens, and a H and P) prior to this biopsy. If the extended pattern clinical biopsy does not meet the inclusion criteria, they will be dropped from enrollment. Final eligibility will be determined after a review of the extended pattern clinical and research biopsy cores by the UCSF Department of Pathology. The purpose of this is to avoid an additional biopsy. If a patient has had a biopsy outside of UCSF, a UCSF pathologist must review the biopsy slides [UCSF pathology review of the most current biopsy slides that indicate malignancy].
- Patients must have 3 serum PSA levels done at least 2 weeks apart over the past year prior to randomization.
- Patients must have PSA ≤10.0, (PSA <15 will be allowed for trial eligibility for patients with benign prostatic hyperplasia or prostatitis), Gleason sum ≤6 with no pattern 4 or 5 histology (if Gleason pattern 4 is seen as a microfocus [< 2mm in length]

this will not exclude the patient from trial eligibility), and clinical stage T1 or T2a. Patients must have no more than 33% of biopsy cores positive (if ≥33% of biopsy cores are positive due to microfoci of adenocarcinoma, this will not exclude the patient from trial eligibility), and no more than 50% of the length of a tumor core involved by carcinoma. These criteria are based on the literature in watchful waiting[59, 60], and are expected to minimize the risk that a patient would develop metastatic disease as a result of participating in the study.

- Life expectancy of greater than 3 months and ECOG performance status ≤2 are required
- Ability to understand and willingness to sign an informed consent document.

Exclusion criteria

- Any prior or concurrent treatment for prostate cancer, including surgery, radiation, hormonal therapy (Lupron, Casodex, Flutamide, Zoladex, Megace, Nilutamide, DES/estrogen), chemotherapy, PC-SPES, or investigational agents.
- Patients with PSA doubling time < three months are ineligible.
- Use of lycopene, fish oil, or any other preparation intended to supplement levels of omega-3 unsaturated fatty acids within 4 weeks of study entry and during the 3-month intervention period.
- Use of Finasteride, Dutasteride, Saw Palmetto or any other herbal/nutritional preparation indicated to affect hormone levels within 4 weeks of study entry and during the 3-month intervention period.
- Use of NSAIDs, Cox-2 inhibitors, and/or aspirin for more than 7 days over the one month prior to study enrollment and during the 3-month intervention period.
- History of allergic reactions attributed to tomatoes, fish, soybean oil, gelatin capsules, or compounds of similar chemical or biologic composition to lycopene (carotenoids) or fish oil.

- Uncontrolled intercurrent illness including, but not limited to, ongoing or active infection, symptomatic congestive heart failure, unstable angina pectoris, cardiac arrhythmia, or psychiatric illness/social situations that would limit compliance with study requirements.

4.3 Inclusion of women and minorities

Since women do not get prostate cancer, they are not eligible for this study. Men of all ethnic groups are eligible to participate. The proposed study population, based on accrual to an existing prostate gene expression study is illustrated in the table below.

Race/Ethnicity

White, not of Hispanic Origin	Black, not of Hispanic Origin	Hispanic	Asian or Pacific Islander	Unknown	Total
63.4%	14%	4.3%	13%	4.3%	100%

4.4 Total number

Assuming independence of genes, 29patients will be randomized to the lycopene intervention, 29to the fish oil intervention and 29 to the control arm. This total evaluable sample size of 87 is sufficient to detect 60% of the intervention arm showing two-fold decrease in *IGF-1* and *Cox-2* gene expression compared with 20% of the control group. An additional 10 patients will be entered for a total planned accrual of 97, assuming a 10% dropout rate (final evaluable target number in each arm is29). (see statistical considerations, below).

4.5 Recruitment

Patients are referred to the program in Urologic Oncology at Mt. Zion hospital for clinical management of prostate cancer. All those who meet the inclusion criteria will be invited to participate at the time of the office consultation. The study will be presented to eligible patients by treating physicians, who will obtain consent from those agreeing to participate.

4.6 Method of treatment assignment and blinding

Patients will be stratified prior to randomization based upon their dietary intake of tomato and fish. Four strata have been defined (low tomato, low fish; low tomato, high fish; high tomato,

low fish; and high tomato, high fish) using the results from a pilot study of 96 subjects. The cutoff for low vs. high tomato-product intake will be four servings/week; the cutoff for low vs. high fish intake will be two servings per week. Patients will be randomized from within each of the 4 strata to one of the 3 study regimens (placebo, lycopene or omega-3) with equal probability. This will ensure that there is a balance of patients based upon current dietary intake among the 3 arms. Treatment assignment will be balanced after every 9 patients are randomized within each stratum. Because the distribution by strata is unknown the block size is small to avoid an imbalance as the study approaches conclusion. As indicated each intervention arm will be compared with the placebo group so it is important to maintain a balance in baseline diet as to lycopene and omega-3 in each of the 3 study arms.

Data on usual intake in this trial will be assessed at baseline by the same dietary survey used in the pilot study.

In the control arm (arm 1), patients will maintain their usual diet and take placebos representing lycopene and fish oil. In the lycopene arm (arm 2), patients will take 30mg of lycopene and a placebo representing fish oil, in the omega-3 arm (arm 3), they will take 3g of fish oil daily, and a placebo representing lycopene.

5 Treatment Plan

5.1 Table of drug administration

Drug	Dosage	Administration vehicle/volume	Route	Instructions	Starting Date	Duration
Lycopene	30mg	15mg softgels	Oral	2 softgels daily	Day 1	90 days
Lycopene Placebo	N/A	Placebo softgel	Oral	2 softgels daily	Day 1	90 Days
Fish oil **EPA=1098 mg DHA=549 mg**	3g	1000mg softgels	Oral	3 softgels daily	Day 1	90 days
Fish Oil Placebo	Not applicable	Placebo Softgel	Oral	3 softgels daily	Day 1	90 days
Multivitamin	Tab	Capsule	Oral	1 daily	Day 1	90 days

5.2 Written description of drug dosing

Patients will take placebo, lycopene or fish oil supplements daily, as oral preparations, on an outpatient basis. The investigational pharmacy at the Cancer Center will maintain an excel

spreadsheet which will record the number and type of placebo, fish oil and lycopene capsules dispensed. In addition, to counter the possibility that different vitamin preparations could affect study results, patients will be asked to cease their usual vitamin intake and use a standard multivitamin (that we will provide). Patients must refrain from taking any other supplements, herbs, or other treatments during the 3-month intervention period.

5.3 Duration of therapy

Treatment will continue for 90 days, or until follow-up biopsy is scheduled (the maximum duration of treatment will be 104 days). During the planning process for this protocol, we met with prostate cancer advocates and support groups. At these town hall meetings, we learned that more than 50% of men eligible for watchful waiting were already taking a variety of nutrition supplements. The men in these groups expressed willingness to forgo their usual regimen for a period of three months, but they felt that continuing to 6 months or 12 months would deter them from participating in the study. Thus, to aid in rapid accrual, we have selected a three month duration for the intervention. Based on other preliminary studies[41], this 90 day period will be sufficient to observe biological changes in target prostate tissue.

Other pertinent info about administration

None.

5.4 Dose Modification

Since the agents studied are of very low toxicity, no dose modifications are anticipated and none are permitted.

6 Agent Formulation and Procurement

The Urologic Oncology investigational pharmacist will be responsible for overseeing production, storage, oversight, and blinding of gel-capsules containing the study doses of lycopene, fish oil, or placebo. We have researched several academic laboratories that measure serum levels of lycopene and omega-3 fatty acid [1, 5, 7, 9, 16, 27, 41, 64-67], and will negotiate a contract based on availability and cost before the trial is initiated.

Lycopene

Chemical name

Psi-psi-carotene

Other names

none

Molecular formula

$C_{40}H_{56}$

MW

536.9

Preparation

15 mg gel capsule

Storage

Room temperature

Stability

<5% degradation by HPLC at 3 years

Route of administration

Oral

Reported adverse events, potential risks

No reported adverse events.

Potential drug interactions

None known.

Procurement

Lycopene will be supplied by LycoRed Natural Products Industries Ltd.

Agent ordering

Through research pharmacy due to placebo control

Agent accountability

Inventory maintained by investigational pharmacy

Fish oil

Chemical name

Omega-3 unsaturated fatty acid

Other names

Docosohexaenoic acid

Molecular formula

22:6,n-3

MW

356.8

Preparation

1g gel capsule

Storage

Room temperature

Stability

Expiration in 06/2008

Route of administration

oral

Reported adverse events, potential risks

Allergic reactions to gelatin capsule, fish. Patients may smell like fish.

Potential drug interactions

Since omega-3 fatty acids alter prostaglandin levels, patients on long-term treatment with NSAIDS, Cox-2 inhibitors, and/or aspirin may have increased bleeding tendency. Patients on these agents are excluded from the trial.

Procurement

Fish Oil will be supplied by Roche Vitamins Inc.

Agent ordering

Through research pharmacy due to placebo control

Agent accountability

Inventory maintained by investigational pharmacy.

7 Dose Modifications

Various doses have been used in previous clinical trials of lycopene and fish oil. Lycopene has been administered in clinical trials at 13.3 mg[30],15mg[16, 27, 29] [31], 25mg[64], and 30mg per day[28, 68]. No patient enrolled in these trials was reported to suffer an adverse effect while taking lycopene or during follow-up. Omega-3-enriched fish oil also has been administered at a variety of doses in the clinical setting as well - 10g for twelve weeks[41], 6g for eight weeks[69], 6g for up to 28 months[70], 5.2g for six weeks[71], 3.4g for six months[72], 1.65g EPA and DHA for two years[73], and 0.85g for 3.5 years[67]. These trials also demonstrated no adverse effect to patients while taking omega-3 fatty acids in the form of fish oil supplements or during follow-up.

We will proceed with giving 30mg of lycopene or 3g of fish oil doses for the MENS study. These doses have been selected based on the minimal and maximal range at which previous

clinical trials have observed physiologic effects and no toxicity for lycopene and fish oil supplementation. We selected an intervention duration of three months based on previous studies by other groups which documented gene expression changes[15, 16, 41]

8 Study Assessments

8.1 Registration

CRA will assess eligibility, and perform block randomization if appropriate. The investigational pharmacist will provide the patient with blinded capsules and a standard multivitamin. Follow up visits will be arranged, and coordinated with the urologist to ensure tissue acquisition at biopsy visits.

8.2 Screening assessments

Baseline assessment—Clinical

Before beginning the study a medical history and physical examination, including a digital rectal exam, will be done. On entry into the study, patients will undergo a limited core biopsy by Dr. Shinohara or another urologist from the UCSF Urologic Oncology Department to procure fresh tissue for genomic analysis. Patients who have already donated core biopsies at the time of diagnosis for the Prostate Cancer Tissue Bank will be asked to participate if they meet the inclusion criteria, but will not need to be re-biopsied. Standard clinical history and physical exam, including digital rectal exam, will be performed, and serum will be assayed for PSA, free PSA, testosterone, estrogen, lycopene, omega-3 fatty acid, omega-6 fatty acid, IGFBP-3 , and IGF-1. Tissue biopsies for research purposes will also be sent for standard pathologic examination. All patients will fill out a survey instrument characterizing their dietary habits, the Willett SFFQ, a validated food questionnaire as well as a food diary to be completed daily for 3 days prior to the biopsy. All patients will also complete a Health and Prostate Cancer Survey at baseline and at 3 months.

In addition to any prostate biopsy obtained above, serum and urine will be obtained and frozen for future analysis. These samples will be used to investigate the presence of any proteins identified in the gene discovery process. There is currently a CHR protocol in place (99394 CHR#H7720-10671-05) for the collection of cancer specimens for the study of molecular changes.

Baseline assessment—Molecular

Tissue - If a patient consents, core needle biopsy tissue will be collected in a standard fashion via transrectal ultrasound guided biopsy. Some tissue will be used immediately for testing. Any remaining tissue will be stored at the UCSF Comprehensive Cancer Center Tissue Bank for possible future research. Samples will only be obtained from patients who give written informed consent. Samples become property of the UCSF Tissue Bank and may not be transferred to another institution. Other investigators will have access to this tissue only with patient consent and through approval of the UCSF Tissue Bank Committee. Tissue samples will receive a unique identifier to protect confidentiality and link clinical outcome to patient samples. Patients may withdraw consent at any time and any unused samples will be destroyed.

Blood - Approximately 36ml of peripheral blood will be collected. Samples will be assigned a unique identifier to protect confidentiality and facilitate data analysis.

Urine - Approximately 20ml of urine will be collected. Samples will be assigned a unique identifier to protect confidentiality and facilitate data analysis. Urine will be given to the Cancer Center Tissue Core Laboratory, and frozen for future study.

An aliquot of total RNA from dissected normal prostate tissue from each core needle biopsy will be directly reverse transcribed and assessed using Taqman quantitative PCR for *IGF-1* and *PTGS2/COX2*[74-76].

cDNA array hybridization: Patient Test and reference pool fluorescent RNA will be mixed with 20 micrograms of Cot-1 DNA and purified away from unincorporated dye using a Qiagen PCR purification spin column. The column eluate will be dried by lyophilization, and dissolved in a hybridization mixture with a final concentration of 3XSSC, 0.3%SDS, 0.05M Hepes pH7.0, and 10mg/ml polyadenylic acid. The hybridization solution will be denatured by incubation at

94C for 4 minutes to denature the DNA, and applied to the slide under a 25x40 mm cover slip (lifter slips, Eerie Scientific). Hybridization reactions will be allowed to proceed at 63.5C for 14-16 hours with slides contained in a Plexiglas chamber (HybeChamber, Genemachines, Redwood City, CA) submerged in a water bath. After hybridization, each slide will be washed in 2X SSC, 0.2% SDS for 5 minutes at 65C in a Coplin jar, followed by 4 minutes in 2X SSC at room temperature on a gently agitating rotator platform, and 1 minute in 0.065X SSC at room temperature. Excess liquid will be removed from slides by spinning at 600 rpm in a Beckman Allegra clinical centrifuge.

Arrays will be imaged with a confocal laser scanning microscope (Axon 4000B). Post image-acquisition data analysis will be performed with Genepix 3.0 software (Axon), and ratio information corrected for background fluorescence will be uploaded into a relational database (NOMAD, Derisi lab, UCSF unpublished) housed on the laboratory Linux web server. NOMAD will then be queried to produce normalized datasets that can be manipulated by other analysis algorithms. There is no consensus on how to normalize array data across groups working in gene expression profiling. Our approach, implemented by NOMAD software is to make the sum of signals in each channel, cy3 and cy5, equal. Though it may be that more up- or down-regulation is present in the cy3 or cy5 channels for a particular nutritional intervention, we assume that averaging across the large number of genes present on each array will allow identification of modulated genes. The identification of *caspase-3* regulation in prostate cancer attests to the practicality of this approach (unpublished data, Haqq lab).

Initial microarray studies will collate sets of named and novel genes that are differentially expressed according to baseline dietary intake quartiles using supervised and unsupervised hierarchical cluster analysis[62] and statistical analysis for microarrays (SAM)[63], a software package that implements a permutation T-test to identify genes up- or down-regulated with a measure of statistical confidence.

We will select particular genes whose expression is correlated with baseline dietary intake for validation of activity of the supplement by an independent assay technique. Quantitative PCR accomplished with the Taqman technology[74-76] is ideally suited to target validation for the MENS study, since the RNA amounts available from clinical core needle biopsies range from 100-600 nanograms. These small amounts of RNA preclude the use of other techniques such as

Northern blot analysis, and are more easily modified for high throughput analysis of several gene targets as compared to RNAse protection – RNAse protection experiments are also cumbersome due to requirements for radioisotope use. The UCSF Comprehensive Cancer Center has a genomics core available to Dr. Haqq's laboratory which will carry out Taqman quantitative PCR[74-76]

8.3 Assessment during the treatment period

Follow-up assessment—Clinical

Standardized Clinical Follow-up Protocol - At the three month follow-up, patients will undergo a standard history and physical exam including digital rectal examination performed by the treating physician. Serum and red blood cells will be drawn for PSA, testosterone, estrogen, lycopene, omega-3 fatty acid, omega-6 fatty acid, IGFBP-3, and IGF-1 levels. Patients will have a second appointment for research purposes only, with a study urologist (Dr. Shinohara or another urologist from the UCSF Urologic Oncology Department) who will obtain four directed prostate core needle samples under ultrasound guidance in order to re-biopsy the areas of tumor and normal prostate identified at the baseline visit to fulfill the study primary outcome measure of detecting gene expression modulation by nutrients. Tissue biopsies for research purposes will also be sent for standard pathologic examination. As obtaining biopsies three months after electing Watchful Waiting is not standard practice, the study will bear costs for the biopsy procedure. In addition to any prostate biopsy obtained above, serum and urine will be obtained and frozen for future analysis. Patients will fill out a second food diary during the second month of treatment intervention and a third food diary prior to their biopsy. These food diaries will be used to collect information about changes in dietary behavior associated with recent diagnosis with prostate cancer, dietary counseling, or participation in this trial. Patients will complete another Health and Prostate Cancer Survey after three months. Patients will also be asked to self-report their compliance with the study treatment, and to return their medicine so that pill counts can be performed. Based on the results of the three-month follow-up visit, serum and pathology tests, the treating physician will assess the patient for disease progression.

Follow-up assessment—Molecular

We will assess compliance and effectiveness of the nutritional supplements by measuring serum levels of lycopene, omega-3 and omega-6 fatty acids at baseline and post-intervention.

All of the procedures for follow-up tissue processing are identical to those for baseline assessment. In addition, we will examine in detail the nutritional effect on gene expression in molecular pathways known to play a critical role in the formation of tumors such as the cell cycle, apoptotic and DNA repair pathways. For this analysis we have implemented automated pathway mapping software from the Conklin laboratory at UCSF. The GenMapp software package (www.genmapp.org) maps gene expression results from arrays onto pathways that can be customized or written entirely by the user, providing graphic output with gene expression ratios color coded on pathway diagrams. Using GenMapp, we have shown specific defects in the apoptotic pathway, the androgen receptor pathway, and the TGF-beta pathway in hormone sensitive and refractory prostate cancer patients.

8.4 Early termination or final visit

Patients may terminate the study at any time for any reason. Patients finishing the 3 month intervention and 21 month monitoring period without progression will be offered standard care for their prostate cancer.

8.5 Additional safety follow-up

None.

8.6 Study table: timing of physical exam, labs, transrectal ultrasound and biopsy

The dietary intervention and molecular analysis will be complete within approximately three months for each patient. We anticipate that it will take up to three years to accrue the target number of patients and conduct the molecular analysis. Patients remaining on the watchful waiting protocol—regardless of assignment to control or intervention arms—will be followed indefinitely according to UCSF standard of care in the Urologic Oncology program.

STUDY TABLE:

	+/-1-4 week(s)	+/-3 months@	+/-6 months	+/-9 months	+/-12 months	Continuing[a]
Office Visit with Vital Signs	X	X	X	X	X	X[a]
TRUS-biopsy	X[b]	X[g]			X[c]	X[a]
PSA	X[d]	X	X	X	X	X[a]
Digital Rectal Exam	X	X	X	X	X	X[a]
Food Questionnaire	X				X	
Health and Prostate QOL	X	X				
Pill Counts		X				
Microarray	X	X				
Serum Testosterone	X	X	X		X	
Serum Estradiol	X	X	X		X	
Serum lycopene	X	X	X		X	
Serum IGFBP-3	X	X	X		X	
Tissue Cox-2	X	X				
Tissue IGF-1	X	X				
Serum IGF-1	X	X	X		X	
Serum Omega3/6 ratio	X	X	X		X	
Placebo or nutritional supplement and MVI administration[e]	X					
Dietary Counseling	X					
Serum banking[f]	X	X	X		X	
Urine[f]	X	X				

	+/-1-4 week(s)	+/-3 months@	+/-6 months	+/-9 months	+/-12 months	Continuing[a]

[a]after the completion of the 3 month intervention period, patients will be referred back to their primary UCSF urologist for further care, which may include the indicated assessments.

[b]patients who have donated prostate needle biopsy tissue within 3 months of study entry to the existing Prostate Cancer Tissue Bank will not undergo the initial TRUS biopsy, since their gene expression pattern will have already been comprehensively assessed, and RNA for the primary study aims banked.

[c]need for the 12 month TRUS biopsy will be determined by the PSA progression criteria in section 9.4. Some patients, specifically those whose PSA doubling time remains > 12 months at the final study visit will not be asked to undergo the 12 month biopsy.

[d] three serum PSAs at least 2 weeks apart over the past year to permit calculation of a PSA doubling time [77] must be available. For those patients without serial PSA measurements, randomization will be delayed until collection of the third PSA. Patients with PSA doubling time < 3 months are ineligible to participate.

[e]Patients will remain on the assigned intervention – placebo, lycopene or fish oil, until the follow-up appointment for transrectal ultrasound and prostate biopsy occurs. Due to scheduling constraints, such as the occurrence of weekends or holidays, it is anticipated that some patients may have to stay on supplement between one to two weeks longer than the literal three month period. Any patient where repeat biopsy cannot be obtained by 14 weeks after beginning intervention will be censored.

[f]Serum and urine are banked at study entry but not used in the primary data acquisition. These resources are anticipated for possible future confirmatory studies if predictors of progression are identified. Patients will initial their consent for these samples at the end of the informed consent form.

[g]The three month research biopsies will be performed at no cost to the participating study subjects.

9 Criteria for Response, Progression, and Relapse

9.1 Definitions

See clinical section 9.4

9.2 Guidelines for evaluation

Not applicable.

9.3 Molecular

Primary – not applicable.

Secondary – not applicable.

9.4 Clinical

We define disease progression as: PSA doubling time within 3 months; any adverse pathological findings at baseline, three, six, nine, or 12 months if extended pattern biopsies are performed with a Gleason sum ≥7 (see exception below); involvement of > 50% of any core; > 1/3 of cores positive; or other incidental evidence of clinical progression (i.e. positive bone scans or lymph node biopsies). Any patient exhibiting progression at any time will be withdrawn from the study and offered standard treatment options. Any patient may also withdraw from the protocol at any time for any reason and be offered standard treatment.

If extended pattern biopsies are performed with a Gleason sum ≥7, but with a pattern 4 seen as a microfocus (< 2mm in length), this will not exclude the patient from continuing on trial.

Regarding the 4 core research biopsies, if involvement of > 50% of any core or > 1/3 of cores are positive, this will NOT exclude the participant from continuing in the study unless a Gleason pattern 4 is seen (if Gleason pattern 4 is seen as a microfocus [< 2mm in length] this will not exclude the patient from continuing on trial).

10 Safety and ethical considerations

10.1 Safety plan – Oversight and Monitoring

The UCSF Comprehensive Cancer Center (UCSFCCC) Data Monitoring Committee (DMC) is responsible for monitoring data quality and patient safety for all UCSFCCC clinical studies. Phase I-III nutrition trials are required to have a data safety and monitoring plan commensurate with patient risk approved by the protocol review committee (PRC). A summary of DMC activities follows:

-Review of all clinical trials conducted at the UCSFCCC for progress and safety

-Review of all adverse events requiring expedited reporting as defined in this protocol

-Review of reports generated by the UCSFCCC data quality control review process

-Submit recommendations for corrective action to the Clinical Research Steering Committee (CRSC)

-Notify the Principal Investigator of the DMC recommendation to the CRSC

-Notify external sites

10.2 Assessment of safety

Assessment of Safety

Toxicity is not expected in the MENS study, and since there is a stopping rule for efficacy, no patient should be harmed by participation in this protocol. Many of the past studies of omega-3 supplementation in humans studied coronary atherosclerosis using angiography. Despite canulation of the arterial system after more than two years of 12-gram per day supplementation, no bleeding complications occurred in this study[70]. Likewise, there are no -expected side effects from lycopene supplementation[41].

Serum PSA and clinical progression will be monitored at visits approximately every three months.

Adverse Events

10.2.2.1 **Adverse Event Reporting (AER)**

10.2.2.2 **Investigational Drug Branch (IDB)**

Investigators are required by Federal Regulations to report serious adverse events as defined in the table below. Investigators are required to notify the Investigational Drug Branch (IDB), Principal Investigator and their Institutional Review Board if a patient has a reportable serious adverse event (follow guidelines in the table below). This study will utilize the Common Toxicity Criteria version 2 to determine the severity of the reaction for adverse event reporting.

Investigators are also required to report secondary malignancies occurring on or following treatment on NCI-sponsored protocols. Reporting of cases of secondary AML/MDS is to be performed using the NCI/CTEP Secondary AML/MDS Report Form. This form should be used in place of the form FDA #3500 (Medwatch). All other secondary malignancies should be reported using the form FDA #3500 (Medwatch).

10.2.2.3 **Expedited reporting for adverse events** (including hospitalization defined below) attributable to investigational agent(s) is required as in Table 1

TABLE 1: Expedited Reporting for Phase 2 and Phase 3 Studies

UNEXPECTED EVENT		EXPECTED EVENT	
GRADES 2 – 3<br>**Attribution of Possible, Probable or Definite**	GRADES 4 and 5<br>**Regardless of Attribution**	GRADES 1 – 3	GRADES 4 and 5<br>**Regardless of Attribution**

Expedited report within 10 working days.<br><br>(Grade 1 Adverse Event Expedited Reporting NOT required.)	Report by phone to IDB within 24 hrs. Expedited report to follow within 10 working days.<br><br>This includes all deaths within 30 days of the last dose of treatment with an investigational agent regardless of attribution.<br><br>Any late death attributed to the agent (possible, probable, or definite) should be reported within 10 working days.	Adverse Event Expedited Reporting NOT required.	Expedited report, including Grade 5 Aplasia in leukemia patients, within 10 working days.<br><br>This includes all deaths within 30 days of the last dose of treatment with an investigational agent regardless of attribution.<br><br>Any late death attributed to the agent (possible, probable, or definite) should be reported within 10 working days.<br><br>Grade 4 Myelosuppression or other Grade 4 events that do not require expedited reporting will be specified in the protocol.

- For **Hospitalization** only - Any medical event equivalent to CTC Grade 3, 4, 5 which precipitated hospitalization (or prolongation of existing hospitalization) must be reported regardless of expected or unexpected and attribution.
- Telephone reports to the Investigational Drug Branch at 301-230-2330 available 24 hours daily (recorder between 5 pm and 9 am EST).
- Expedited reports are to be submitted using AdEERS or the paper templates available at http://ctep.cancer.gov/ The NCI Guidelines for expedited adverse event reporting are also available at this site.
- A list of agent specific expected adverse events can be found in the Appendix A-1.
- Includes reporting of all deaths within 30 days of the last dose of treatment regardless of attribution.

10.2.2.4 **Adverse Events not Requiring Expedited Reporting**

The occurrence of any of the following adverse events does not require expedited reporting unless unusually severe and/or causing prolonged hospitalization:

Hypotension, fatigue (lethargy, malaise, asthenia), fever, rash/desquamation, nausea, vomiting, SGOT (AST) serum glutamic oxaloacetic transaminase),

SGPT (ALT) (serum glutamic pyruvic transaminase), acidosis, hyperglycemia, hypocalcemia, syncope (fainting), headache, myalgia (muscle pain), apnea, and hypoxia.

10.2.2.5 Institutional Review Board (IRB) Reporting

The reporting of adverse reactions described in the tables above is in addition to and does not supplant the reporting of adverse events as part of the report of the results of the clinical trial, e.g. study summary forms or cooeprative group data reporting forms.

Baseline Medical Condition

Uncontrolled intercurrent illness including, but not limited to, ongoing or active infection, symptomatic congestive heart failure, unstable angina pectoris, cardiac arrhythmia, or psychiatric illness/social situations that would limit compliance with study requirements is an exclusion criterion. Due to the minimal toxicity of the agents under study, all other patients are eligible, with ECOG status <= 2.

Laboratory Tests/Other Evaluations

Serum PSA, testosterone, Estradiol, lycopene and omega-3/6 ratio will be determined. Details of other molecular tests are given in the study design sections.

Vital Signs

Vital signs will be obtained at each patient visit. Abnormal vital signs will be reported as defined in the common toxicity criteria above.

10.3 Adverse event reporting

See section 10.2

10.4 Consent

Verbal and written consent for participation will be obtained by the patient's physician. Additionally, patients will be asked to provide consent for each tissue donation, even if they have already agreed to participate in a previous tissue donation for the project.

10.5 Confidentiality, risks, and protection of subjects

Risks/discomforts

Conservative management of early prostate cancer has been reported as a successful option for select patients. However, it remains an experimental approach, and it is possible that patients may experience disease progression in excess of that which would have occurred with primary active treatment. Furthermore, patients may experience adverse psychological impact by deferring active treatment for known cancer. Patients wishing to end participation in the study for this reason may do so at any time and proceed with active treatment. Additional prostate biopsy entails slight discomfort and minimal additive risk of bleeding and/or infection. Anesthetic risk is minimized by the use of local nerve block.

Compensation for injury

Any injury resulting from prostate biopsies for the purposes of this study will be compensated per the standard policies of the University of California and/or the Veterans Administration, as appropriate.

Alternatives

Patients are free to decline participation with no impact whatsoever on their patient status at UCSF or its affiliates; they may also withdraw from the study at any time. Patients declining or withdrawing will be offered standard definitive treatment for prostate cancer as appropriate by their physician.

Costs and reimbursement

The National Cancer Institute will supply the lycopene, fish oil and placebo supplements. The cost of office visits, laboratory tests and biopsies, (except for the three month research biopsy) will be billed to patients and/or insurance companies in standard fashion. Every effort will be made to obtain authorization from patients' insurance companies for all treatments, tests, and doctors office appointments. Insurance companies have sometimes refused to pay for the costs of standard treatment for patients on research studies, in which case patients will be responsible for

all costs. Patients should check with the doctors and/or financial counselor if they have questions regarding the costs of treatment and your eligibility for payment programs.

There will be no reimbursement to subjects for participation.

Confidentiality

Specimens will be assigned accession numbers that are unique for the project, and no reference is made to any patient identifier. In order to determine clinical endpoints, we must review patient's records. Every precaution will be taken to preserve patient confidentiality – access to patient identifiers is limited to the database manager, and principal investigators. The identifier will be recorded in a logbook in addition to the signed consent forms and stored in a locked cabinet in the principal investigator's office. No laboratory results will be reported back to the patient or his physician, and no patient contact is made unless specifically requested by the patient. No individual patient will be identified in any publication.

10.6 Ethics

For men who elect watchful waiting, there is no generally accepted standard of care. This study will assess whether nutrition supplements lead to desirable molecular changes. The use of a placebo controlled trial is ethical in this circumstance, since there is no data to support that denying active intervention with either lycopene or fish oil would lead to harmful cancer progression.

10.7 Compliance with laws and regulations

This protocol will be subjected to institutional review board scrutiny. Regulations for consent and adverse event reporting will be followed.

11 Ancillary Therapy

11.1 What is allowed during treatment

No other treatments for prostate cancer (surgery, radiation, hormonal therapy, chemotherapy, investigational agents, or herbal preparations) will be allowed during the three months of the primary study period. Patients will be asked to refrain from taking additional dietary supplements beyond those provided by protocol.

12 Removal of Patients from Protocol Therapy

Patients will be removed from protocol therapy for clinical progression defined above. They will then be offered standard active therapy for their prostate cancer.

13 Statistical Considerations

Patients satisfying eligibility requirements will complete a dietary questionnaire prior to randomization.

Patients will not be taking lycopene or omega-3 supplements, but other dietary sources of tomato lycopene or fish oil could possibly influence the assessment of outcome for this study. For example, it might be that an excess of tomato-consuming patients would be placed in the control arm, confounding the assessment of gene expression in response to lycopene. Therefore, patients will be stratified into four groups (low tomato/low fish, low tomato/high fish, high tomato/low fish, and high tomato/high fish) and block-randomized to placebo, lycopene, or fish oil, within these strata. This will avoid the chance occurrence of confounding due to usual intake of fish or tomato products (e.g. unbalanced patient features among the study arms). Cutoffs for the baseline dietary strata are based on the medians of intake from a **completed** independent pilot study on 96 prostate cancer patients from the source population at the UCSF Mt. Zion Cancer Center, who completed the Willett Semi-Quantitative Food Frequency Questionnaire. The median for total tomato consumption was 4 servings/week; for fish consumption it was two servings/week.

We will assess changes in serum lycopene and omega-3 fatty acid pre- and post-intervention to assess compliance and the physiologic significance of the supplementation doses.

For our primary analysis, changes in gene expression for each of the two nutrient interventions will be compared with that of the control arm. The two intervention arms will not be compared with each other. The primary outcome measure will be at least a two fold up decrease in gene expression of *IGF-1* or *COX-2* at three-months relative to the initial biopsy for a given patient (both normalized to levels of a control housekeeping gene, *β-glucuronidase*). To compare the intervention and control groups, we define an important difference to be a 40% difference in the proportion of patients on the intervention arm compared with the placebo arm who satisfy the primary outcome measure defined above (e.g. 20% in control vs. 60% in intervention showing at least a two-fold decrease in mRNA level). Fisher's exact test for proportions will be used to determine if *IGF-1* or *COX-2* mRNA levels are statistically significantly different between the groups.

For the secondary specific aims (aims 1.2 through 1.4 defined above), we will consider a microarray dataset with measurements for 30,000 genes. For this exploratory investigation genes will be treated as being independent so as to first identify all possible genes that may indicate an effect due to the nutritional supplement (Aim 1.2) or to other dietary components (each considered as a binomial variable) (Aim 1.3). Fisher's exact test will be used to determine which genes result in differences in proportions with up or down regulated genes between groups. No adjustment for multiple comparisons will be made. Also, after all patients have had the potential to be followed for at least one year, they will be classified as to whether they had experienced disease progression within 1 year from protocol entry. The same exploratory techniques described above first using the baseline gene expression profile will be carried out to identify genes that may be associated with disease progression (Aim 1.4). Even though any nutritional supplement will be stopped at 3 months from the start of protocol therapy and the duration of any effect is unknown, this analysis will be repeated using the 3-month gene expression profile to identify the effects of any gene that might have been modified by protocol treatment. To account for genes with highly variable expression regardless of study arm, genes whose expression levels change two-fold in more than 20% of men on placebo (comparing three-month to baseline) will

be excluded from subsequent consideration. This might exclude possible predictors of outcome, but if there is variability within the placebo group then the results may be biased. This refining process will isolate potential predictors of nutritional change when assessing the primary as well as the secondary outcomes. Standard microarray gene expression software algorithms will facilitate analyses to determine if there are individual genes or clusters of genes associated with either supplement, dietary component or disease progression[62, 63] . Fisher's exact test will be used to determine which genes result in statistically significant differences between the groups.

To evaluate the primary study aim, 29 patients will be randomized to the lycopene intervention, 29 to the fish oil intervention and 29 to the control arm. Analysis of 87 evaluable patients is sufficient to detect a 40% difference in the proportion of patients with a 2-fold decrease in *IGF-1* mRNA levels with lycopene or in the *Cox-2* gene with fish oil when compared with the control. The total accrual of 97 patients takes into consideration a 10% dropout rate by the time of the three-month biopsy. The level of significance for a directional test is set at .025 because the control group will be compared with each of the two intervention groups. The power of each test is set at 81% (nQuery).

Descriptive statistics (mean, confidence intervals, median, proportions) will be calculated to characterize the patients' baseline disease features (e.g. age, Gleason score, PSA) and self-reported nutritional dietary data within each arm. Differences between each of the intervention arms and the placebo arm will be assessed using a chi-square statistic for categorical variables, a t-statistic for continuous variables and the Mann-Whitney statistic for distributions. Estimates of the probability of remaining free of disease progression will be calculated using the Kaplan-Meier product limit method. The log rank test will be used to compare tumor progression between each supplement group and the control (Aim 1.5).

The randomized clinical trial design of this study limits the potential for confounding due to other factors, however this will be investigated by comparing the distribution of clinical parameters and other diet/lifestyle factors as well as changes after 3 months of study treatment among the three arms. Unequally distributed factors would only bias the results if they also

correlate with changes in gene expression. If necessary, the Mantel-Haenszel test for a stratified analysis would be performed in evaluating the primary study aim.

Interim Analysis:

An interim analysis for efficacy will be performed after 10 patients have been accrued to each of the 3 regimens and had the potential to be followed for 12 months from entry. With watchful waiting approximately 25% of patients will experience disease progression within 12 months of diagnosis. If more than 5 of the 10 patients on either supplement arm experience disease progression by 12 months from protocol entry, the study will be closed to further accrual. This will indicate that the 95% lower bound at 12 months is greater than 25% (30.4%). It is anticipated that this analysis will occur 15 months after the study is opened when approximately 45 patients have been entered on study. A statistician in the UCSF Comprehensive Cancer Center not associated with the design or final analysis will conduct this interim analysis.

14 Qualifications of investigators

Dr. June Chan is Assistant Professor in the department of Epidemiology and Biostatistics and has studied the effects of diet, nutritional supplements and clinical therapy on prostate cancer incidence and progression for several years, initially at the Harvard School of Public Health, and now at UCSF. Dr. Peter Carroll is co-director of the Genitourinary Oncology Program at UCSF and is Chairman of the Department of Urology, with an active practice. Dr. Christopher Haqq is an Assistant Adjunct Professor in Urology and has been studying microarray technology and representative mRNA amplification for several years in the lab of Frank McCormick, director of the cancer center and a pioneer in the area of signal transduction and viral anticancer therapy. He has also been studying gene expression in hormone sensitive and hormone refractory men with prostate during the last year, and has identified candidate genes characterizing each of these disease stages. He is also the principle investigator for the National Cancer Institute, National Institutes of Health grant R01 CA 101042-01 “Molecular Effects of Nutrition Supplements in the Prostate.” Dr. Katsuto Shinohara is Professor in the Department of Urology. He is internationally recognized as an expert in transrectal ultrasound and core needle biopsy sampling. Dr.

Christopher Kane is an Associate Professor in the Department of Urology. Dr. Matthew Cooperberg is a Fellow in Genitourinary Oncology. Dr. Maxwell Meng is an Assistant Professor in the Department of Urology. Dr. Kirsten Greene is a Clinical Instructor in the Department of Urology. Vivian Weinberg, PhD is Principal Biostatistician.

15 References

1. Di Mascio, P., S. Kaiser, and H. Sies, *Lycopene as the most efficient biological carotenoid singlet oxygen quencher.* Arch Biochem Biophys, 1989. **274**(2): p. 532-8.
2. Ascherio, A., et al., *Correlations of vitamin A and E intakes with the plasma concentrations of carotenoids and tocopherols among American men and women.* J Nutr, 1992. **122**(9): p. 1792-801.
3. Kaplan, L.A., et al., *Reference ranges of retinol, tocopherols, lycopene and alpha- and beta- carotene in plasma by simultaneous high-performance liquid chromatographic analysis.* Clin Physiol Biochem, 1987. **5**(6): p. 297-304.
4. Kaplan, L.A., et al., *Simultaneous, high-performance liquid chromatographic analysis of retinol, tocopherols, lycopene, and alpha- and beta-carotene in serum and plasma.* Methods Enzymol, 1990. **189**: p. 155-67.
5. Clinton, S.K., et al., *cis-trans lycopene isomers, carotenoids, and retinol in the human prostate.* Cancer Epidemiol Biomarkers Prev, 1996. **5**(10): p. 823-33.
6. Giovannucci, E., et al., *Intake of carotenoids and retinol in relation to risk of prostate cancer.* J Natl Cancer Inst, 1995. **87**(23): p. 1767-76.
7. Gann, P.H., et al., *Lower prostate cancer risk in men with elevated plasma lycopene levels: results of a prospective analysis.* Cancer Res, 1999. **59**(6): p. 1225-30.
8. Vogt, T.M., et al., *Serum lycopene, other serum carotenoids, and risk of prostate cancer in US Blacks and Whites.* Am J Epidemiol, 2002. **155**(11): p. 1023-32.
9. Chen, L., et al., *Oxidative DNA damage in prostate cancer patients consuming tomato sauce- based entrees as a whole-food intervention.* J Natl Cancer Inst, 2001. **93**(24): p. 1872-9.
10. Kucuk, O. and D.P. Wood, Jr., *Re: Response of hormone refractory prostate cancer to lycopene.* J Urol, 2002. **167**(2 Pt 1): p. 651.
11. Matlaga, B.R., et al., *Response of hormone refractory prostate cancer to lycopene.* J Urol, 2001. **166**(2): p. 613.
12. Kedishvili, N.Y., et al., *Evidence that the human gene for prostate short-chain dehydrogenase/reductase (PSDR1) encodes a novel retinal reductase (RalR1).* J Biol Chem, 2002. **29**: p. 29.
13. Kotake-Nara, E., et al., *Carotenoids affect proliferation of human prostate cancer cells.* J Nutr, 2001. **131**(12): p. 3303-6.
14. Kotake-Nara, E., et al., *Acyclo-retinoic acid induces apoptosis in human prostate cancer cells.* Anticancer Res, 2002. **22**(2A): p. 689-95.
15. Mucci, L.A., et al., *Are dietary influences on the risk of prostate cancer mediated through the insulin-like growth factor system?* BJU Int, 2001. **87**(9): p. 814-20.
16. Kucuk, O., et al., *Phase II randomized clinical trial of lycopene supplementation before radical prostatectomy.* Cancer Epidemiol Biomarkers Prev, 2001. **10**(8): p. 861-8.
17. Miyake, H., et al., *Overexpression of insulin-like growth factor binding protein-5 helps accelerate progression to androgen-independence in the human prostate LNCaP tumor model through activation of phosphatidylinositol 3'-kinase pathway.* Endocrinology, 2000. **141**(6): p. 2257-65.

18. Klein, R.D. and S.M. Fischer, *Black tea polyphenols inhibit IGF-I-induced signaling through Akt in normal prostate epithelial cells and Du145 prostate carcinoma cells.* Carcinogenesis, 2002. **23**(1): p. 217-21.
19. Sies, H. and W. Stahl, *Lycopene: antioxidant and biological effects and its bioavailability in the human.* Proc Soc Exp Biol Med, 1998. **218**(2): p. 121-4.
20. Breinholt, V., et al., *Dose-response effects of lycopene on selected drug-metabolizing and antioxidant enzymes in the rat.* Cancer Lett, 2000. **154**(2): p. 201-10.
21. Clark, R.M., et al., *A comparison of lycopene and astaxanthin absorption from corn oil and olive oil emulsions.* Lipids, 2000. **35**(7): p. 803-6.
22. Bohm, V. and R. Bitsch, *Intestinal absorption of lycopene from different matrices and interactions to other carotenoids, the lipid status, and the antioxidant capacity of human plasma.* Eur J Nutr, 1999. **38**(3): p. 118-25.
23. Mellert, W., et al., *Thirteen-week oral toxicity study of synthetic lycopene products in rats.* Food Chem Toxicol, 2002. **40**(11): p. 1581-8.
24. Paetau, I., et al., *Chronic ingestion of lycopene-rich tomato juice or lycopene supplements significantly increases plasma concentrations of lycopene and related tomato carotenoids in humans.* Am J Clin Nutr, 1998. **68**(6): p. 1187-95.
25. Porrini, M., P. Riso, and G. Testolin, *Absorption of lycopene from single or daily portions of raw and processed tomato.* Br J Nutr, 1998. **80**(4): p. 353-61.
26. Paetau, I., et al., *Carotenoids in human buccal mucosa cells after 4 wk of supplementation with tomato juice or lycopene supplements.* Am J Clin Nutr, 1999. **70**(4): p. 490-4.
27. Hughes, D.A., et al., *Effects of lycopene and lutein supplementation on the expression of functionally associated surface molecules on blood monocytes from healthy male nonsmokers.* J Infect Dis, 2000. **182 Suppl 1**: p. S11-5.
28. Neuman, I., H. Nahum, and A. Ben-Amotz, *Reduction of exercise-induced asthma oxidative stress by lycopene, a natural antioxidant.* Allergy, 2000. **55**(12): p. 1184-9.
29. Hininger, I.A., et al., *No significant effects of lutein, lycopene or beta-carotene supplementation on biological markers of oxidative stress and LDL oxidizability in healthy adult subjects.* J Am Coll Nutr, 2001. **20**(3): p. 232-8.
30. Corridan, B.M., et al., *Low-dose supplementation with lycopene or beta-carotene does not enhance cell-mediated immunity in healthy free-living elderly humans.* Eur J Clin Nutr, 2001. **55**(8): p. 627-35.
31. Olmedilla, B., et al., *A European multicentre, placebo-controlled supplementation study with alpha-tocopherol, carotene-rich palm oil, lutein or lycopene: analysis of serum responses.* Clin Sci (Lond), 2002. **102**(4): p. 447-56.
32. Augustsson, K., et al., *A prospective study of intake of fish and marine fatty acids and prostate cancer.* Cancer Epidemiol Biomarkers Prev, 2003. **12**(1): p. 64-7.
33. Terry, P., et al., *Fatty fish consumption and risk of prostate cancer.* Lancet, 2001. **357**(9270): p. 1764-6.
34. Norrish, A.E., et al., *Prostate cancer risk and consumption of fish oils: a dietary biomarker- based case-control study.* Br J Cancer, 1999. **81**(7): p. 1238-42.
35. Amiano, P., et al., *Very-long-chain omega-3 fatty acids as markers for habitual fish intake in a population consuming mainly lean fish: the EPIC cohort of Gipuzkoa. European Prospective Investigation into Cancer and Nutrition.* Eur J Clin Nutr, 2001. **55**(10): p. 827-32.
36. Rose, D.P. and J.M. Connolly, *Effects of fatty acids and eicosanoid synthesis inhibitors on the growth of two human prostate cancer cell lines.* Prostate, 1991. **18**(3): p. 243-54.
37. Karmali, R.A., et al., *The effects of dietary omega-3 fatty acids on the DU-145 transplantable human prostatic tumor.* Anticancer Res, 1987. **7**(6): p. 1173-9.
38. Tjandrawinata, R.R., R. Dahiya, and M. Hughes-Fulford, *Induction of cyclo-oxygenase-2 mRNA by prostaglandin E2 in human prostatic carcinoma cells.* Br J Cancer, 1997. **75**(8): p. 1111-8.

39. Chaudry, A.A., et al., *Arachidonic acid metabolism in benign and malignant prostatic tissue in vitro: effects of fatty acids and cyclooxygenase inhibitors.* Int J Cancer, 1994. **57**(2): p. 176-80.
40. Morcos, N.C. and K. Camilo, *Acute and chronic toxicity study of fish oil and garlic combination.* Int J Vitam Nutr Res, 2001. **71**(5): p. 306-12.
41. Aronson, W.J., et al., *Modulation of omega-3/omega-6 polyunsaturated ratios with dietary fish oils in men with prostate cancer.* Urology, 2001. **58**(2): p. 283-8.
42. Chan, D.C., et al., *Effect of atorvastatin and fish oil on plasma high-sensitivity C-reactive protein concentrations in individuals with visceral obesity.* Clin Chem, 2002. **48**(6 Pt 1): p. 877-83.
43. Grimble, R.F., et al., *The ability of fish oil to suppress tumor necrosis factor alpha production by peripheral blood mononuclear cells in healthy men is associated with polymorphisms in genes that influence tumor necrosis factor alpha production.* Am J Clin Nutr, 2002. **76**(2): p. 454-9.
44. Olsen, S.F., et al., *Randomised clinical trials of fish oil supplementation in high risk pregnancies. Fish Oil Trials In Pregnancy (FOTIP) Team.* Bjog, 2000. **107**(3): p. 382-95.
45. Smit, E.N., et al., *Fish oil supplementation improves docosahexaenoic acid status of malnourished infants.* Arch Dis Child, 2000. **82**(5): p. 366-9.
46. Jemal, A., et al., *Cancer statistics, 2003.* CA Cancer J Clin, 2003. **53**(1): p. 5-26.
47. Boyle, P., P. Maisonneuve, and P. Napalkov, *Geographical and temporal patterns of incidence and mortality from prostate cancer.* Urology, 1995. **46**(3 Suppl A): p. 47-55.
48. Quinlan, D.M., A.W. Partin, and P.C. Walsh, *Can aggressive prostatic carcinomas be identified and can their natural history be altered by treatment?* Urology, 1995. **46**(3 Suppl A): p. 77-82.
49. Steinberg, G.D., G.T. Bales, and C.B. Brendler, *An analysis of watchful waiting for clinically localized prostate cancer.* J Urol, 1998. **159**(5): p. 1431-6.
50. Wei, J.T., et al., *Comprehensive comparison of health-related quality of life after contemporary therapies for localized prostate cancer.* J Clin Oncol, 2002. **20**(2): p. 557-66.
51. Albertsen, P., *Unraveling the epidemiology of prostate cancer.* J Urol, 1997. **157**(6): p. 2223-4.
52. Johansson, J.E., et al., *Fifteen-year survival in prostate cancer. A prospective, population-based study in Sweden.* Jama, 1997. **277**(6): p. 467-71.
53. Lu-Yao, G.L. and S.L. Yao, *Population-based study of long-term survival in patients with clinically localised prostate cancer.* Lancet, 1997. **349**(9056): p. 906-10.
54. Koppie, T.M., et al., *Patterns of treatment of patients with prostate cancer initially managed with surveillance: results from The CaPSURE database. Cancer of the Prostate Strategic Urological Research Endeavor.* J Urol, 2000. **164**(1): p. 81-8.
55. Zietman, A.L., et al., *Conservative management of prostate cancer in the prostate specific antigen era: the incidence and time course of subsequent therapy.* J Urol, 2001. **166**(5): p. 1702-6.
56. Derksen, J.E., A.V. Kshirsagar, and J.L. Mohler, *Psychological consequences of observation versus radical prostatectomy for clinically localized prostate cancer three and ten years later [abstract].* J Urol, 2002. **167**(4S): p. 1347a.
57. Vollmer, R.T., et al., *The dynamics of prostate specific antigen during watchful waiting of prostate carcinoma: a study of 94 Japanese men.* Cancer, 2002. **94**(6): p. 1692-8.
58. Wilt, T.J. and M.K. Brawer, *Early intervention or expectant management for prostate cancer. The Prostate Cancer Intervention Versus Observation Trial (PIVOT): a randomized trial comparing radical prostatectomy with expectant management for the treatment of clinically localized prostate cancer.* Semin Urol, 1995. **13**(2): p. 130-6.
59. Choo, R., et al., *Feasibility study: watchful waiting for localized low to intermediate grade prostate carcinoma with selective delayed intervention based on prostate specific antigen, histological and/or clinical progression.* J Urol, 2002. **167**(4): p. 1664-9.

60. Carter, H.B., et al., *Expectant management of nonpalpable prostate cancer with curative intent: preliminary results.* J Urol, 2002. **167**(3): p. 1231-4.
61. Epstein, J.I., P.C. Walsh, and H.B. Carter, *Dedifferentiation of prostate cancer grade with time in men followed expectantly for stage T1c disease.* J Urol, 2001. **166**(5): p. 1688-91.
62. Eisen, M.B., et al., *Cluster analysis and display of genome-wide expression patterns.* Proc Natl Acad Sci U S A, 1998. **95**(25): p. 14863-8.
63. Tusher, V.G., R. Tibshirani, and G. Chu, *Significance analysis of microarrays applied to the ionizing radiation response.* Proceedings of the National Academy of Sciences of the United States of America, 2001. **98**: p. 5116-5121.
64. Richelle, M., et al., *A food-based formulation provides lycopene with the same bioavailability to humans as that from tomato paste.* J Nutr, 2002. **132**(3): p. 404-8.
65. Harvei, S., et al., *Prediagnostic level of fatty acids in serum phospholipids: omega-3 and omega-6 fatty acids and the risk of prostate cancer.* Int J Cancer, 1997. **71**(4): p. 545-51.
66. Godley, P.A., et al., *Biomarkers of essential fatty acid consumption and risk of prostatic carcinoma.* Cancer Epidemiol Biomarkers Prev, 1996. **5**(11): p. 889-95.
67. *Dietary supplementation with n-3 polyunsaturated fatty acids and vitamin E after myocardial infarction: results of the GISSI-Prevenzione trial. Gruppo Italiano per lo Studio della Sopravvivenza nell'Infarto miocardico.* Lancet, 1999. **354**(9177): p. 447-55.
68. Chen, L., et al., *Oxidative DNA damage in prostate cancer patients consuming tomato sauce-based entrees as a whole-food intervention.* J Natl Cancer Inst, 2001. **93**(24): p. 1872-9.
69. Luo, J., et al., *Moderate intake of n-3 fatty acids for 2 months has no detrimental effect on glucose metabolism and could ameliorate the lipid profile in type 2 diabetic men. Results of a controlled study.* Diabetes Care, 1998. **21**(5): p. 717-24.
70. Sacks, F.M., et al., *Controlled trial of fish oil for regression of human coronary atherosclerosis. HARP Research Group.* J Am Coll Cardiol, 1995. **25**(7): p. 1492-8.
71. Tinker, L.F., et al., *(n-3) fatty acid supplementation in moderately hypertriglyceridemic adults changes postprandial lipid and apolipoprotein B responses to a standardized test meal.* J Nutr, 1999. **129**(6): p. 1126-34.
72. Eritsland, J., et al., *Long-term metabolic effects of n-3 polyunsaturated fatty acids in patients with coronary artery disease.* Am J Clin Nutr, 1995. **61**(4): p. 831-6.
73. von Schacky, C., et al., *The effect of dietary omega-3 fatty acids on coronary atherosclerosis. A randomized, double-blind, placebo-controlled trial.* Ann Intern Med, 1999. **130**(7): p. 554-62.
74. Ginzinger, D.G., *Gene quantification using real-time quantitative PCR. An emerging technology hits the mainstream.* Exp Hematol, 2002. **30**(6): p. 503-12.
75. Aldape, K., D.G. Ginzinger, and T.E. Godfrey, *Real-time quantitative polymerase chain reaction: a potential tool for genetic analysis in neuropathology.* Brain Pathol, 2002. **12**(1): p. 54-66.
76. Lamarcq, L., et al., *Measurements of human papillomavirus transcripts by real time quantitative reverse transcription-polymerase chain reaction in samples collected for cervical cancer screening.* J Mol Diagn, 2002. **4**(2): p. 97-102.
77. Pound, C.R., et al., *Natural history of progression after PSA elevation following radical prostatectomy.* JAMA, 1999. 281(17): p. 1591-97.
